# Dissecting the genetic basis of resistance to *Soil-borne cereal mosaic virus* (SBCMV) in durum wheat by bi-parental mapping and GWAS

**DOI:** 10.1007/s00122-024-04709-7

**Published:** 2024-09-02

**Authors:** Martina Bruschi, Matteo Bozzoli, Claudio Ratti, Giuseppe Sciara, Ellen Goudemand, Pierre Devaux, Danara Ormanbekova, Cristian Forestan, Simona Corneti, Sandra Stefanelli, Sara Castelletti, Elena Fusari, Jad B Novi, Elisabetta Frascaroli, Silvio Salvi, Dragan Perovic, Agata Gadaleta, Concepcion Rubies-Autonell, Maria Corinna Sanguineti, Roberto Tuberosa, Marco Maccaferri

**Affiliations:** 1 Department of Agricultural and Food Sciences (DISTAL), Alma Mater Studiorum – Università di Bologna, 40127 Bologna, Italy; 2 S.A.S. Florimond-Desprez Veuve and Fils, BP41, 59242 Cappelle-en-Pévèle, France; 3 Federal Research Centre for Cultivated Plants, Institute for Resistance Research and Stress Tolerance, Julius Kühn-Institut (JKI), Erwin-Baur-Str. 27, 06484 Quedlinburg, Germany; 4 Department of Soil, Plant and Food Science (Di.S.S.P.A.), University of Bari ‘Aldo Moro’, 70126 Bari, Italy

## Abstract

*Soil-borne cereal mosaic virus* (SBCMV), the causative agent of wheat mosaic, is a *Furovirus* challenging wheat production all over Europe. Differently from bread wheat, durum wheat shows greater susceptibility and stronger yield penalties, so identification and genetic characterization of resistance sources are major targets for durum genetics and breeding. The *Sbm1* locus providing high level of resistance to SBCMV was mapped in bread wheat to the 5DL chromosome arm (Bass in Genome 49:1140–1148, 2006). This excluded the direct use of *Sbm1* for durum wheat improvement. Only one major QTL has been mapped in durum wheat, namely *QSbm.ubo-2B,* on the 2BS chromosome region coincident with *Sbm2*, already known in bread wheat as reported (Bayles in HGCA Project Report, 2007). Therefore, *QSbm.ubo-2B* = *Sbm2* is considered a pillar for growing durum in SBCMV-affected areas. Herein, we report the fine mapping of *Sbm2* based on bi-parental mapping and GWAS, using the Infinium 90 K SNP array and high-throughput KASP®. Fine mapping pointed out a critical haploblock of 3.2 Mb defined by concatenated SNPs successfully converted to high-throughput KASP® markers coded as *KUBO*. The combination of *KUBO-27*, *wPt-2106-ASO/HRM*, *KUBO-29,* and *KUBO-1* allows unequivocal tracing of the *Sbm2*-resistant haplotype. The interval harbors 52 high- and 41 low-confidence genes, encoding 17 cytochrome p450, three receptor kinases, two defensins, and three *NBS-LRR* genes. These results pave the way for *Sbm2* positional cloning*. *Importantly, the development of *Sbm2* haplotype tagging KASP® provides a valuable case study for improving efficacy of the European variety testing system and, ultimately, the decision-making process related to varietal characterization and choice.

**Supplementary Information:**

The online version contains supplementary material available at 10.1007/s00122-024-04709-7.

## Introduction

*Soil-borne cereal mosaic virus* (SBCMV) is a furovirus affecting the production of both tetraploid durum (*Triticum turgidum* var. *durum* Desf.) and hexaploid bread wheat (*Triticum aestivum* L.) across Europe, especially in France, Italy, Germany, Poland, and Denmark (Budge et al. 2008; Kanyuka et al. 2004; Maccaferri et al. 2011a; Rubies-Autonell et al. 2003). SBCMV was initially considered the European strain of *soil-borne wheat mosaic virus* (SBWMV), a member of the genus *Furovirus* which affects mainly wheat crops in U.S., and later recognized by the International Committee on Taxonomy of Viruses as a separate species (Kanyuka et al. 2004).

Fields heavily infected with SBCMV have been shown to suffer a yield reduction of ca. 40–50% in susceptible winter wheat cultivars in the UK and up to 70% in highly susceptible durum wheat in Italy (Clover et al. 1999; Kühne 2009; Vallega and Rubies Autonell 1985; Vallega et al. 2003).

The name “soil-borne” is due to the infection mechanism which takes advantage of the plasmodiophorid vector *Polymyxa graminis*, a soil inhabitant fungal-like endoparasitic protist slime mold, endoparasite of wheat. As a vector, *P. graminis* produces resting spores that are easily spread mainly by water, wind, and machinery. SBCMV nests inside the resting spores of the vector during periods of adverse environmental conditions and long rotations of non-host crops, persisting and accumulating in soil for decades. When conditions reverse to favorable for disease development (e.g., presence of susceptible hosts and high soil moisture, particularly in the fall, when extended periods of high soil moisture favor the movement of *Polymyxa graminis*), the virus is released from the resting spores and, once vectored through zoospores, enters root hair cells (Kühne 2009). From there, the virus can reach the aerial part of the plant and infect the leaf tissue, causing symptoms ranging from mild mottling to severe leaf mosaic. Moreover, vigor and plant height are also affected, with susceptible cultivars showing stunting (Kim et al. 2017; Kühne 2009; Perovic et al. 2009; Roberts 2014).

Due to the nature and persistence of the natural inoculum, agronomic practices like crop rotation or delayed sowing are mostly ineffective once the soil is infected. Chemical defense is neither effective nor environmentally friendly. As a consequence, the only possible option is the development and deployment of resistant cultivars (Kanyuka et al. 2003; Ordon et al. 2009).

The first and most effective SBCMV resistance locus identified in the bread wheat germplasm was *Sbm1*, located on chromosome 5DL (Bass et al. 2006). Resistance was detected in bread wheat cv. Cadenza by assessing a doubled haploid population obtained from the cross Avalon (susceptible cultivar) × Cadenza (resistant cultivar). Resistance, mainly of “translocation type” preventing the infection to spread from the roots to the stem and leaves (Kanyuka et al. 2004; Ordon et al. 2009), was found to be controlled by a major locus named *Sbm1* mapped to the long arm of chromosome 5D tagged by microsatellite *Xbarc110* and *Xwmc765* in a region of approximately 17 cM (Bass et al. 2006). Due to the relevance of *Sbm1* for hexaploid wheat breeding, it has been recently fine mapped (Liu et al. 2020) and confined in the 546,651,779—547,273,461-bp interval of Chinese Spring RefSeq v1.0 pseudomolecule (International Wheat Genome Sequencing Consortium (IWGSC), 2018), harboring 17 candidate genes, with *pto-interacting protein 1* (*PTI1*, TraesCS5D01G531200) identified as the most promising one. After Bass et al. (2006), another major QTL named *Sbm2* was mapped in the same Avalon × Cadenza population by Bayles et al. (2007) in chromosome arm 2BS. *Sbm2* acts in a distinct and complementary way to *Sbm1*, suggesting the possibility to pyramid these two sources of resistance in bread wheat. Taken together, these two major *Sbm* loci enable breeding for bread wheat varieties with valuable SBCMV resistance.

Conversely, durum wheat lacks the D genome; thus, *Sbm1* resistance locus is not directly exploitable for durum wheat breeding. The potentially higher impact of SBCMV on grain yield loss and the lack of known sources of resistance in durum wheat as compared to hexaploid wheat prompted the search for valuable sources of resistance in the durum wheat germplasm. Using a RIL population from Meridiano (resistant) × Claudio (moderately susceptible), Maccaferri et al. (2011a) identified *QSbm.ubo-2B,* a major QTL for SBCMV resistance in durum wheat located in the distal telomeric region of chromosome 2BS, close to the *wPt-2106* DArT® marker and three SSR markers (X*wmc661-Xgwm210-Xbarc35*). The large proportion of variance explained (PVE) by this locus (55.8–68.0% for ELISA and up to 91.6% for symptom severity) suggested *QSbm.ubo-2B* as a potentially major target for durum wheat breeding, marker-assisted selection, and QTL cloning. *QSbm.ubo-2B* was confirmed in a durum wheat panel (Maccaferri et al. 2011a) and in a second mapping population, Simeto (susceptible) × Levante (resistant) with a PVE of 60–70% for symptom severity. Meta-QTL analysis mapped *QSbm.ubo-2BS* on a two cM-wide CI centered to *wPt-2106*, proximal to X*wmc661-Xgwm210-Xbarc35,* which allowed for the first molecular assay to trace the QTL resistant allele. Based on the tetraploid wheat consensus map (Maccaferri et al. 2015) and meta-QTL analysis, *QSbm.ubo-2BS* (Maccaferri et al. 2012) was mapped in a region coincident with the *Sbm2* locus in bread wheat (*QSbm.ubo-2BS* = *Sbm2*). This makes *QSbm.ubo-2BS* = *Sbm2* a valuable target for basic and applied research aimed at its fine mapping and positional cloning.

Currently, the use of single-nucleotide polymorphisms (SNPs, Yuan et al. 2014) has become more efficient, cheaper, and robust than other types of markers and related sequencing techniques in both bread and durum wheat, thanks to the development of species-specific SNP arrays like the Illumina Infinium *iSelect* 90 K wheat SNP array (Cavanagh et al. 2013; Wang et al. 2014). Maccaferri et al. (2015) developed a consensus map for tetraploid wheat harboring 30,144 markers, with high-density gene-derived SNPs as useful anchor points to isolate target loci. SNPs abundance in the wheat genome, together with the development of high-throughput fluorescence-based marker assays, like KASP® (Kompetitive Allele Specific PCR, Semang et al. 2014), makes them ideal for fine mapping and MAS breeding purposes.

The publication of the durum wheat reference cultivar Svevo (Maccaferri et al. 2019) allowed us to easily switch from the genetic to the physical map, accelerating both fine mapping and candidate gene identification. Genome-wide association studies (GWAS) coupled with dense SNPs arrays enable efficient exploitation of large phenotypic datasets to search for and fine map QTLs (Korte and Farlow 2013).

The present work aimed at fine mapping *QSbm.ubo-2BS* using a combination of mapping populations and GWAS. The development of KASP® markers was pursued with the double objective of providing genic markers anchored to the durum wheat Svevo genome assembly suitable for fine mapping of *Sbm2* and, at the same time, assays ready to implement MAS. These results were integrated with candidate genes, identified within the QTL region projected upon the durum wheat cv. Svevo, hexaploid wheat cv. Chinese Spring, and the wild emmer wheat Zavitan (*Triticum turgidum* ssp. *dicoccoides,* Körn. Ex Asch. & Graebn.) reference genomes. Moreover, the results of an extensive survey of the *QSbm.ubo-2BS* = *Sbm2*-resistant/susceptible haplotype distribution in durum wheat panels grown in Europe and worldwide are reported.

## Materials and methods

### Plant materials

#### Mapping populations and cultivated germplasm panels

A recombinant inbred line (RIL) population developed by the University of Bari (Gadaleta et al. 2009) composed of 3,075 lines obtained from cv. Svevo (hereafter referred to as Sv, resistant) and Ciccio (hereafter referred to as *Cc*, susceptible) was used for *Sbm2* fine mapping. Svevo harbors the *Xwmc661*-*Xgwm210*-*Xbarc35*-*wPt-2106* resistance tagging haplotype at *Sbm2* while Ciccio carries the susceptible haplotype, similar to Simeto (Maccaferri et al. 2011a). Cultivar Svevo was sequenced, assembled, and annotated at gold-standard level (Maccaferri et al. 2019). Ciccio is adapted to Southern Mediterranean conditions and is a derivative of Valnova founder, highly susceptible to SBCMV. A second population includes 181 RILs developed by Produttori Sementi Bologna SpA from a cross between cv. Meridiano (hereafter referred to as Mr, resistant) and Claudio (hereafter referred to as Cl, susceptible). The Mr × Cl RIL population was described in Maccaferri et al. (2011a, 2012).

The cultivated durum wheat panels herein considered provide a representation of the cultivated germplasm worldwide spanning a breeding period from 1914/1920 to 2020. A total of 549 varieties/breeding lines included in this study. A first panel, referred as the "UNIBO Durum Panel" (Maccaferri et al. 2011b), was used to perform GWAS and assess linkage disequilibrium extent in the *Sbm2* region and the repeatability and information content of diagnostic Illumina SNP markers converted to fluorescent-based high-throughput KASP® markers. The UNIBO Durum Panel included 258 breeding lines and cultivars released from 1970 to early 2000 from Italy, Spain, CIMMYT, ICARDA, North America, Southwestern USA, France, Austria, and Australia (Maccaferri et al. 2011b). This panel was field phenotyped for SBCMV response over three years. A wider panel including 291 more recent and diverse European varieties was assembled and analyzed in the framework of the Horizon 2020 project "InnoVar, next-generation variety testing for improved cropping on European farmland" and other sources. The complete panel was used to investigate the distribution of SBCMV-resistant and SBCMV-susceptible haplotypes worldwide and across breeding decades.

### Molecular analysis

#### Enrichment of the Sbm2 region with KASP® markers

Based on the consensus map of Maccaferri et al. (2015) and Svevo genome sequence, KASP® assays were developed from 16 Illumina SNPs previously mapped in the *Sbm2* region. Features of these SNPs are reported in Table 1. Primers were designed using PolyMarker (http://Polymarker.tgac.ac.uk) followed by manual curation. Probe sequences tagged with fluorescent dyes FAM and HEX were added to the 5′-end of A and B primers, respectively. KASP® marker development protocol is described in Method S1.

**Table 1:** Summary information with regard to the Illumina Infinium *iSelect* 90 K wheat SNPs chosen to develop polymorphic KASP® markers in the *Sbm2* confidence interval. Illumina 90 K wheat SNPs ID code, SNP marker name, newly developed KASP® marker name, genetic position in the durum wheat consensus map, physical location in durum wheat cv. Svevo genome, and polymorphism in durum / tetraploid recombinant inbred lines. Markers are listed from the distal -5’- to the proximal -3’- 2BS chromosomal region

SNP marker ID code	SNP marker name	KASP® marker	Consensus map position (cM)^a^	Svevo genome assembly position (bp)^b^	Polymorphisms in RIL maps^c^
IWB61884	RAC875_rep_c109471_154	KUBO-015*	7.9	9,766,826	Lt × MG_5323, Cs × Ld, Mr × Cl, Mh × Cr, Sv × Cc
IWB73347	Tdurum_contig76118_145	KUBO-012*	8.4	9,986,862	Lt × MG_5323, Cs × Ld, Mr × Cl, Mh × Cr, Sv × Cc, Sv × Zv
IWB11421	BS00085748_51	KUBO-026*	11.6	13,011,332	Lt × MG_5323, Cs × Ld, Mr × Cl, Sm × Lv, Mh × Cr, Sv × Cc, Sv × Zv
IWB23029	Excalibur_c1787_1037	KUBO-029*	11.6	15,643,691	Cs × Ld, Mr × Cl, Sm × Lv, Sv × Cc
IWB42660	Kukri_c22513_1780	KUBO-032	11.6	13,011,658	Lt × MG_5323, Cs × Ld, Mr × Cl, Sm × Lv, Mh × Cr, Sv × Cc, Sv × Zv
IWB28973	Excalibur_c8093_82	KUBO-001*	12.2	16,235,907	Cs × Ld, Mr × Cl, Mh × Cr, Sv × Cc
IWB45152	Kukri_c41556_619	KUBO-002	12.2	15,713,309	Cs × Ld, Mr × Cl, Mh × Cr, Sv × Cc
IWB41644	Kukri_c16758_443	KUBO-035	12.2	16,183,711	Sm × MC, Cs × Ld, Mr × Cl, Mh × Cr, Sv × Cc
IWB8328	BS00043055_51	KUBO-003*	12.3	15,805,908	Lt × MG_5323, Cl × Ld, Mr × Cl, Sm × Lv, Mh × Cr, Sv × Cc
IWB29097	Excalibur_c841_609	KUBO-040*	12.3	16,185,445	Sm × MC, Cs × Ld, Mr × Cl, Mh × Cr, Sv × Cc
IWB35524	IAAV8700	KUBO-041*	12.3	16,184,325	Sm × MC, Cs × Ld, Mr × Cl, Mh × Cr, Sv × Cc
IWB23330	Excalibur_c19499_948	KUBO-004	12.4	16,957,139	Cs × Ld, Mr × Cl, Sv × Cc
IWB24939	Excalibur_c30167_531	KUBO-005*	12.4	16,954,167	Sm × MC, Cs × Ld, Mr × Cl, Mh × Cr, Sv × Cc
IWB6204	BS00010318_51	KUBO-006*	19.0	24,308,711	Mr × Cl, Sm × Lv, Mh × Cr, Sv × Cc, Sv × Zv
IWB10512	BS00070900_51	KUBO-008*	19.0	24,313,744	Mr × Cl, Sm × Lv, Mh × Cr, Sv × Cc, Sv × Zv
IWB8390	BS00045163_51	KUBO-010	19.3	24,798,904	Lt × MG_5323, Cs × Ld, Sm × Lv, Mh × Cr

cM, (= centiMorgans) are reported as cumulative genetic distances on chromosome 2B according to the consensus map from Maccaferri et al. (2015)

^a^Genetic map distances estimated based on the tetraploid wheat consensus map published in Maccaferri et al. (2015)

^b^Physical order on chromosome 2BS from distal (5’) to proximal (3’) side based on the Svevo genome RefSeq genome assembly published in Maccaferri et al. (2019)

^c^Lt = Latino, MG_5323 = MG5323, Cs = Colosseo, Ld = Lloyd, Mr = Meridiano, Cl = Claudio, Mh = Mohawk, Cr = Cocorit69, Sv = Svevo, Cc = Ciccio, Zv = Zavitan, Sm = Simeto, Lv = Levante, MC = Molise Colli. The corresponding genetic maps have been reported in Maccaferri et al. (2015)

^*^: codominant KASP® marker

#### Genome-wide association study on the UNIBO Durum Panel

The UNIBO Durum Panel was phenotyped in a highly infected field nursery located in Cadriano, Bologna, Italy (44°35′N 11°27′E). The propagule soil content in the field nursery was progressively increased, maintained high, and evenly spread in the nursery soil by repeatedly adopting a wheat-to-wheat rotation with the highly susceptible durum cv. Grazia. This ensured spreading of virulent *P. graminis* spores as clusters of resting spores in root tissue debris or infective propagules directly released in soil and evenly distributed throughout the field.

Phenotypic data were obtained from unreplicated, augmented design field trials over three years (2005, 2007, 2010). Cvs. Svevo, Ciccio, Meridiano, and Claudio were used as common checks across blocks (five blocks per experimental field). Broad-sense heritability was calculated with the R package *heritability* (Kruijer et al. 2015).

Recorded data consisted of visual symptom severity/scores (SEV) in 2005 (single date at end of tillering/first node stage, according to Zadoks et al. (1974), 2007 (four dates), and 2010 (single date) and ELISA values (ELISA, indicating virus concentration in the leaves) in 2007 (two dates) and 2010 (single date). SEV was recorded according to a 0–5 scale modified from Vallega and Rubies Autonell (1985) where: 0 = no symptoms, 0.1–1.5 = slight symptoms, 1.51–2.5 = mild mottling and stunting, 2.51–3.5 = mottling and stunting, 3.51–4.5 = severe mottling and stunting, 4.51–5.0 = plants killed by virus. ELISA assay was performed as in Maccaferri et al. (2012).

Single-plot data were spatially adjusted according to a moving average based on the double adjacent plots. One representative score per year was chosen for further analysis based on correlation among dates, average infection level, and cultivar discrimination power. Dates considered were April 22, 2005, March 6, 2007, and April 7, 2010, for SEV, March 14, 2007, and April 7, 2010, for ELISA. Phenotyping was carried out only for years with nurseries showing an infection level of SEV ≥ 3.00 in susceptible parents and checks throughout the field nursery. Overall infection levels were also concomitantly monitored by ELISA (ELISA ≥ 0.70). Consequently, 2006, 2008, and 2009 nurseries were not considered for further analysis.

Adjusted visual scores and ELISA values were used to calculate best linear unbiased estimates (BLUEs), based on a mixed linear model including genotypes as fixed and all other factors as random (Bates et al. 2014).

GWAS was performed in Tassel 5 (Bradbury et al. 2007) based on genotypic data from Illumina 90 K SNPs, newly developed KASP® markers, *wPt-2106* and visual score and ELISA adjusted BLUES. Genotypic data were converted to *hapmap* format, keeping molecular markers with MAF ≥ 5%, missing data ≤ 50% and by removing genotypes with missing data ≥ 50%. Missing SNPs were subsequently imputed with Beagle v5.4 (Browning and Browning 2022).

A kinship matrix was calculated with all non-redundant SNP markers identified in Haploview 4.2 (Gabriel et al. 2002) with the tagger function set to *r*^2^ = 1.0. Kinship based on identity-by-state (IBS) similarities among accessions was calculated in TASSEL. Each marker was evaluated for association with phenotypic data using a mixed linear model (MLM; Bradbury et al. 2007) which included the kinship matrix (MLM + K). A second round of analysis was carried out using the marker most associated with *Sbm2* region as covariate to highlight additional QTL peaks other than the major one on chromosome 2B. *IWB6584* was chosen as the *Sbm2* tagging marker to be used as covariate.

#### Fine mapping of the Sbm2 region in bi-parental recombinant inbred lines

Aiming to detect new informative recombinant lines in the *Sbm2* confidence region, the complete Svevo × Ciccio RIL set (Sv × Cc RILs, 3,075 lines) was genotyped with the two KASP® markers flanking the *QSbm.ubo-2BS* region, *KUBO-13* and *KUBO-9,* located at the distal (5′) and proximal (3′) sides of the QTL, respectively. The Sv × Cc RILs selected through MAS were sown in two seasons (2015/2016 and 2016/2017) in a field experiment aimed at assessing their SBCMV response in Cadriano (Bologna, Italy, 44°35′N 11°27′E). Further genotyping was performed with seven markers located within the *KUBO-13*/*KUBO-9* interval (i.e., *KUBO-1*, *KUBO-3*, *KUBO-27*, *KUBO-29*, *KUBO-40*, *KUBO*-*41,* and *wPt-2106,* reported based on their distal-to-proximal chromosomal order) with the aim of precisely tracing the recombination events.

An additional set of 30 RILs from the Meridiano × Claudio RIL population (*Mr* × *Cl*, Maccaferri et al. 2011a) with recombinations between *KUBO-3* and *KUBO-9* were selected for detailed *KUBO* genotyping. For each population, genotypic data were used to construct a genetic map was assembled using JoinMap v. 4 (Van Ooijen 2006). Marker order and mapping distances between markers were calculated using the maximum likelihood algorithm and Haldane’s mapping function. The two maps were compared to check for the order of KASP® markers in the region flanked by *KUBO-13* and *KUBO-9*. Once marker order was determined, genotypic and phenotypic data were integrated to define the restricted interval where *QSbm.ubo-2BS* was most probably located. Mapping and association analysis were carried out in Windows QTL Cartographer v. 2.5 by single-marker analysis (Wang et al. 2012). Interval mapping (IM) analysis was also carried out to calculate the LOD score in the interval between *KUBO-13* and *KUBO-9*. The threshold value was set at LOD 3.0 (rounded) via permutations. Walk speed was set at 0.5 cM.

#### Comparison of marker order in the Sbm2 region among genomes

**Fig. 1:**
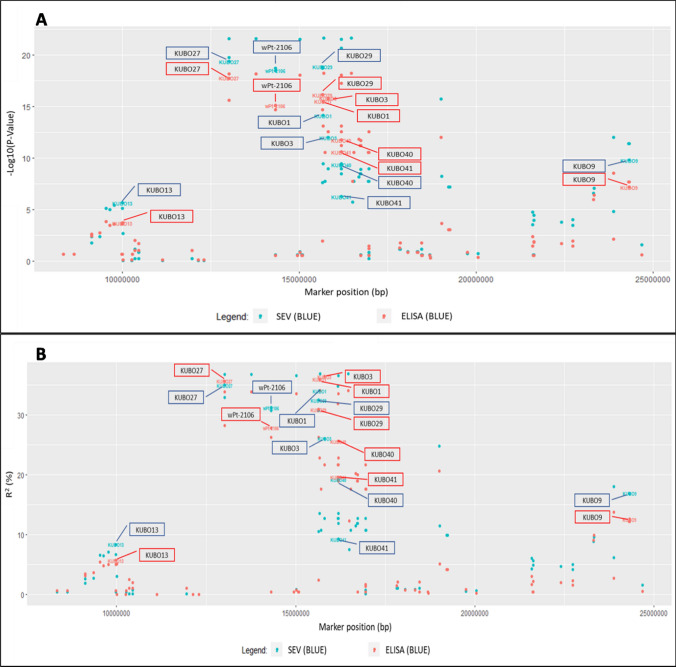
SBCMV response GWAS association results for markers in the *Sbm2* region on chromosome 2BS. Minus log_10_(*P* panel A) and *R*^2^ (%, panel B) GWAS analysis results based on the UNIBO Durum Panel performed with visual score (SEV, shown in dark blue) and ELISA (shown in pale red) BLUEs of field data collected in 2005 (SEV), 2007 (SEV and ELISA), and 2010 (SEV and ELISA) in Cadriano (Bologna, Italy). Marker’s positions are from the Svevo reference genome assembly (Maccaferri et al. 2019). Newly developed KASP® *KUBO* is shown in the picture

**Fig. 2:**
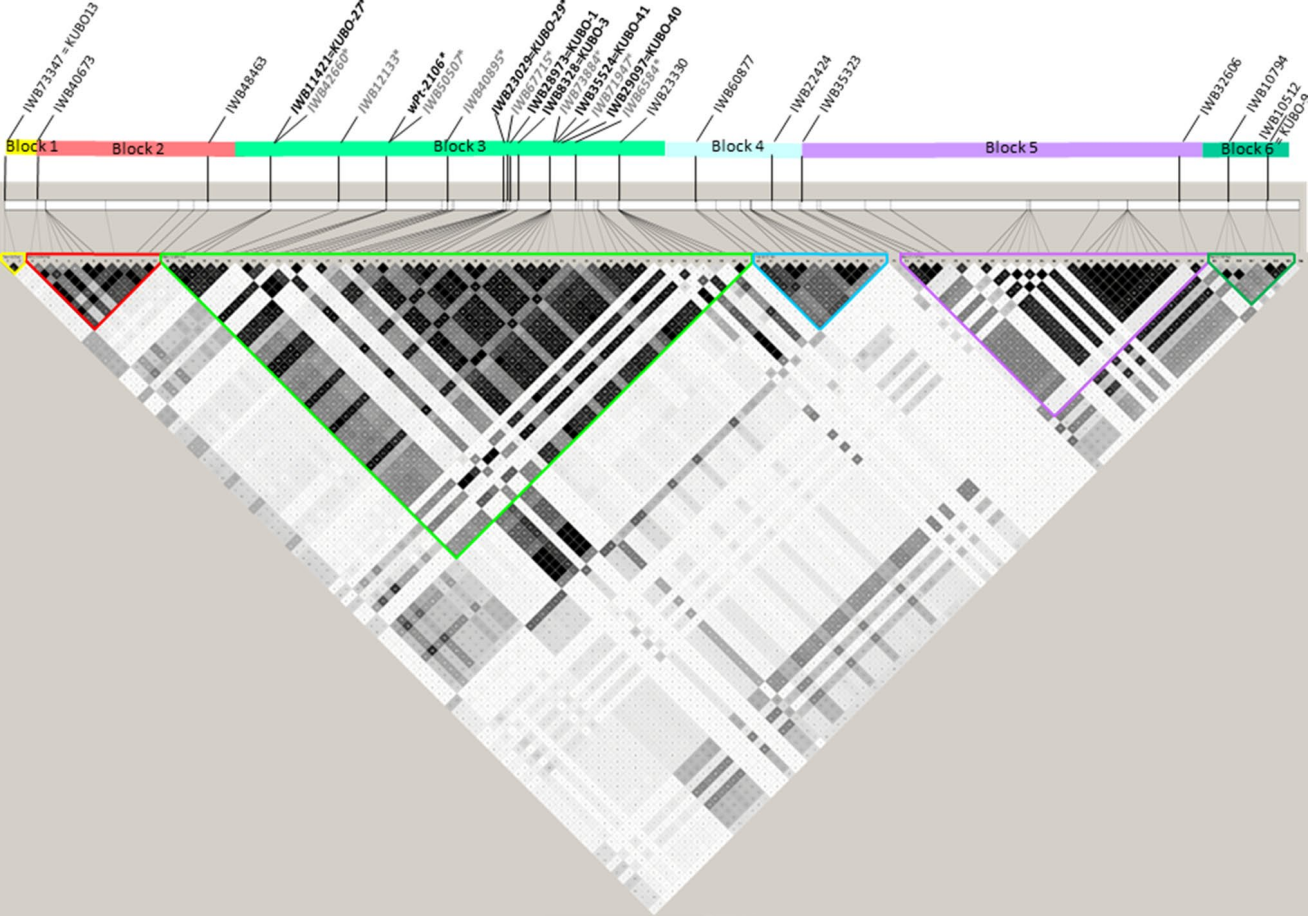
Local linkage disequilibrium (LD) matrix based on pairwise *r*^2^ values in the *KUBO13-KUBO9* chromosome 2BS interval harboring *Sbm2*. The *r*^2^ matrix has been generated in *Haploview* (Barrett et al. 2005). Color-labeled bars represent six adjacent linkage disequilibrium blocks *(hapblocks*) defined based on Gabriel et al. (2002) method

The marker order between *IWB73347* (= *KUBO-13*, position: 9,986,862 bp) and *IWB10512* (= *KUBO-9* position: 24,313,794 bp) was compared between Svevo, Chinese Spring (CS, IWGSC RefSeq V2.1; Zhu et al. 2021) and Zavitan (WEW_v2.0; Zhu et al. 2019) (Figs. 1, 2**, **S5, S6, and S7).

Marker physical positions were determined by BLAST analysis while co-linearity among intervals was compared using *MUMmer4* (Marçais et al. 2018) and manual curation as described in Method S3.

#### Projection of the Sbm2 support interval into the Svevo physical map for candidate gene identification

The *KUBO-27* to *KUBO-40* interval (Figs. 1, 2**, **S5, S6, and S7) was considered to retrieve annotated genes from the Svevo genome reference assembly (Maccaferri et al. 2019). Gene functions were recovered using the functional annotation program *AHRD*, considering the longer transcript as the representative one. GO terms (GO Slim) in *R Bioconductor* (version 3.4) package *GOstats* (Falcon and Gentleman 2007; R Core Team 2017) were used to categorize genes according to their molecular functions and biological processes. Gene intervals were explored with *gProfiler* software (Raudvere et al. 2019). Functional profiling was computed extracting GO molecular functions and biological processes from all the genes of the interval.

#### Sbm2 haplotypes present in the UNIBO Durum Panel

**Fig. 3:**
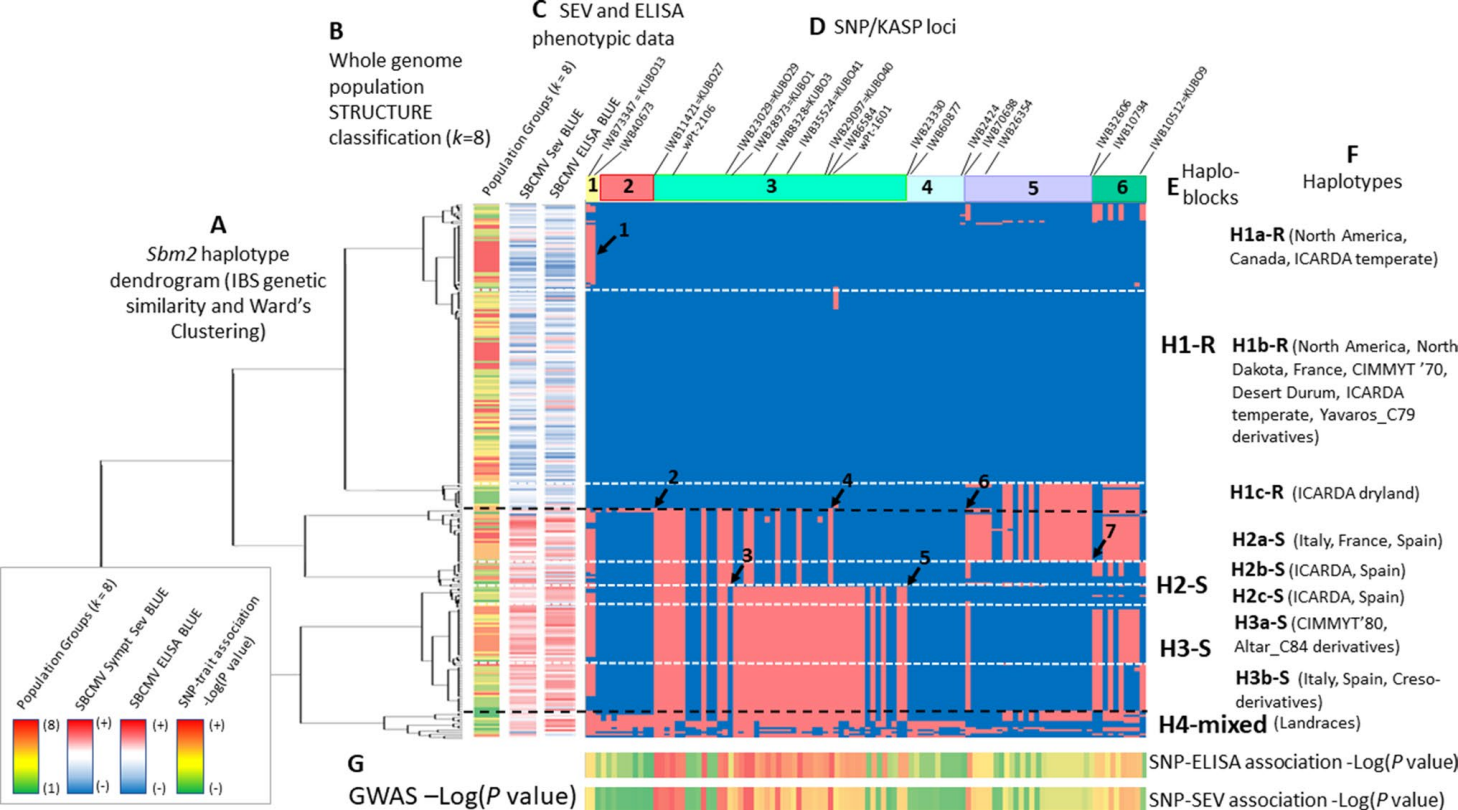
UNIBO Durum Panel local haplotypes in the *KUBO13-KUBO9* chromosome 2BS interval harboring *Sbm2*. Haplotype-based UPGMA identity-by-state IBS genetic similarity was used to group durum panel genotypes and landraces (panel **A**). Each genotype was associated with population structure Q file at *Q* = 8 (**B**) and with SEV and ELISA phenotypic data (**C**). Critical SNP and KUBO KASP® markers are shown in (**D**). Haploblocks detected in Haploview are shown in (**E**) according to Fig. 5. Nucleotide-based haplotypes were represented based on a two-color scheme in panel (**F**). The SNP allele from the resistant parents (Meridiano, Levante, Neodur) was considered as the main leading allele and was colored dark blue while the alternative SNP allele was colored pale red. GWAS minus log_10_ (*P*-value) of SNP-ELISA and SNP-SEV are shown as heat bars in panel (**G**). Critical recombination events are reported with black arrows and numbered from 1 to 7

Based on GWAS results obtained from the Durum Panel UNIBO, the region most associated with *Sbm2* was identified as coincident with the haploblock containing markers *KUBO-27*, *KUBO-29*, *KUBO-1*, *KUBO-3*, *KUBO-41,* and *KUBO-40* (listed from distal to proximal region of chromosome 2BS, Figs. 1, 2, S5, S6, and S7). UNIBO Durum Panel SNP genotype calls in the *Sbm2* region in the *KUBO-13* to *KUBO-9* interval. This interval was used to inspect the local *Sbm2* specific SNP haplotype distribution patterns in cultivars/breeding lines based graphically on a two-color mode (Fig. 3). In the *Sbm2* region, the SNP alleles belonging to the resistant parents/founders Levante, Meridiano, and Neodur (showing identity by descent, IBD, in the region) were dark blue colored while the alternative alleles were pale red colored**.** Additionally, the local *Sbm2* haplotypes depicted in Fig. 3 were clustered based on local genetic similarities (simple matching genetic similarity algorithm, GS, also corresponding to identity by state, IBS) and Ward’s clustering algorithm. Simple matching GS were computed in Tassel 5 and Ward clustering in *pheatmap* R package as in Kolde and Kolde (2015). Linkage disequilibrium among local SNPs and linkage blocks were determined using Haploview (Barrett et al. 2005), defining haploblocks by the algorithm developed by Gabriel et al. (2002). Box plots of SEV and ELISA phenotypes were produced for each haplotype detected at this block.

## Results

### KASP® marker design and validation

Fine mapping of loci of breeding interest and downstream diagnostic (haplotype-based) marker-assisted selection require the development of locally dense molecular marker maps to saturate the confidence interval regions. Therefore, in the case of *Sbm2*, sequences of 16 Illumina Infinium 90 K wheat SNPs mapped in the *Sbm2* region by multiple tetraploid mapping populations (Maccaferri et al. 2015) were deployed to develop high-throughput, fluorescence-based KASP® assays, subsequently used to genotype both RILs and breeding germplasm panels for fine mapping.

Nineteen out of the 35 tested assays (*KUBO-1, KUBO-3, KUBO-5, KUBO-6, KUBO-8, KUBO-9, KUBO-12, KUBO-13, KUBO-14, KUBO-15, KUBO-16, KUBO-17, KUBO-26, KUBO-27, KUBO-29, KUBO-31, KUBO-38, KUBO-40,* and *KUBO-41,* reported based on map order, from the distal 5′-end to the proximal 3′-end region of the QTL on chromosome arm 2BS) proved to be functional, highly repeatable, and codominant. These are ideal for genotyping, resulting in a 54.2% SNP-to-KASP® conversion rate (Illumina SNP and KASP® sequences in Table 1 and Table S1a, respectively).

*KUBO-13* and *KUBO-9*, positioned at the two borders of *Sbm2* region 2.5 cM upstream and 8.1 cM downstream of *wPt-2106,* respectively, were selected to identify recombinants in the Sv × Cc RILs (Table 1 and Fig. S5).

The DArT® marker *wPt-2106,* close to the *Sbm2* peak LOD region, was sequenced and converted to two PCR-based assays, one based on allele-specific (ASO) primers and detectable through horizontal gel electrophoresis assay, and the other based on the high-throughput high-resolution melting technique (Table S1b). Detailed information is reported in Method S1 while Fig. S1 shows PCR assay results for *wPt-2106* DArT marker converted to *wPt-2106* Allele-Specific Oligonucleotide (ASO), *wPt-2106* High-Resolution Melting (HRM, Wittwer et al. 2003).

The newly developed KASP® markers were used to genotype the UNIBO Durum Panel and on RIL populations and validated by comparison with the former Illumina 90 K SNP genotypic calls. KASP® diagnostic marker accuracy results are reported in Table S2 while their amplification plots are shown in Fig. S2.

### GWAS based on the UNIBO Durum Panel

The UNIBO Durum Panel was used as an independent resource to fine map *Sbm2*and to validate the newly developed KASP® markers (genotyping information reported in Method S2). The UNIBO Durum Panel was field evaluated across three years in a dedicated nursery in Cadriano, Bologna, Italy for both visual symptom severity (SEV) and virus content in the leaves (ELISA), as reported in Table S3. SEV and ELISA traits were highly related (Figure S3), with an *r* value of 0.69.

**Fig. 4:**
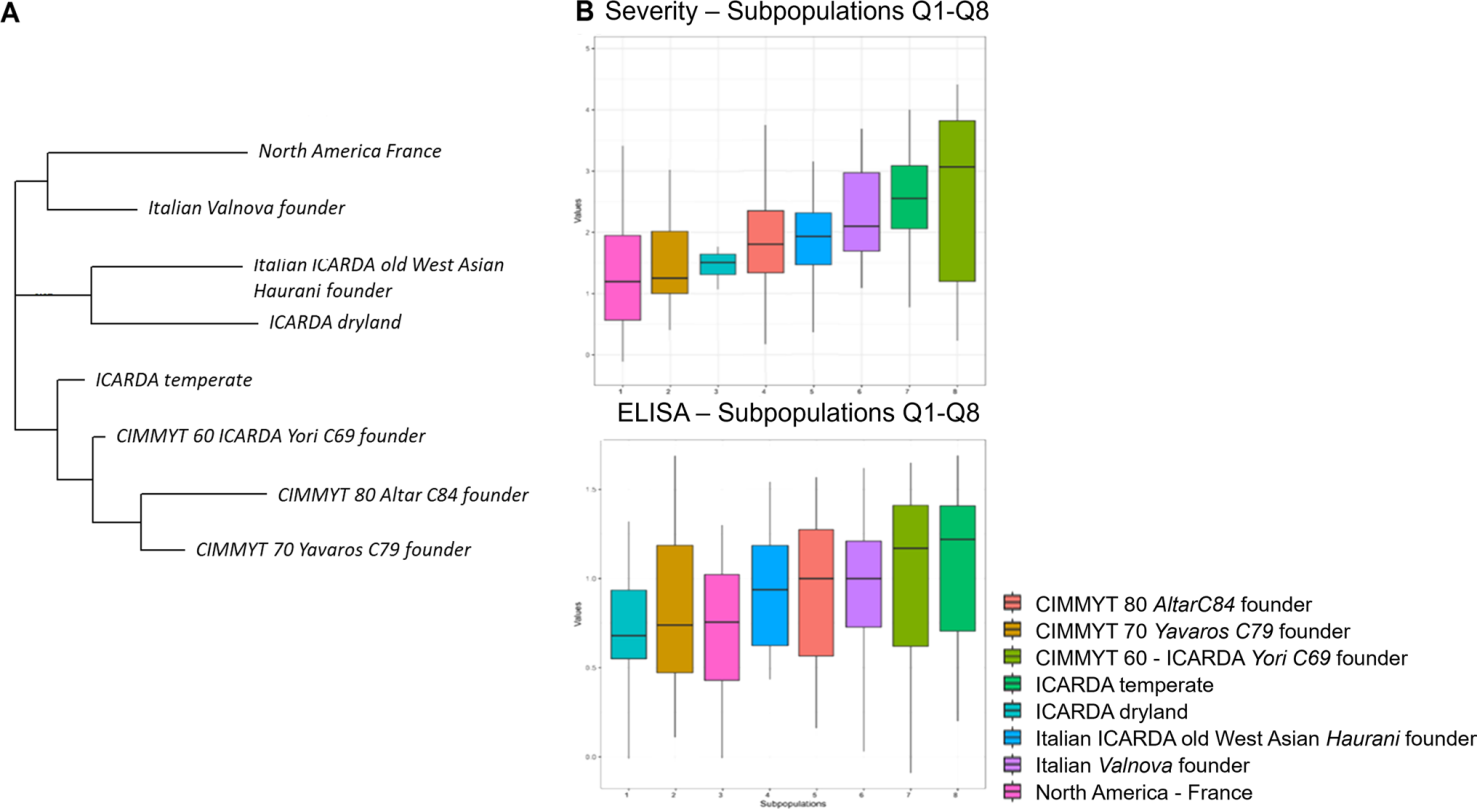
Genetic population structure and SBCMV response phenotypic distributions of varieties and breeding lines included in the UNIBO Durum Panel. **A** neighbor joining tree of the main eight subpopulation groups detected in the UNIBO Durum Panel based on Illumina 90 K SNP. **B** Box-plot phenotypic distributions of UNIBO Durum Panel ELISA and SEV BLUES across years according to the eight subpopulation groups. The molecular-based genetic similarity matrix (= kinship matrix) was obtained using the identity-by-state (IBS) genetic similarity algorithm

The UNIBO Durum Panel showed a population genetic structure including eight well-defined groups of varieties/breeding lines corresponding to the main breeding programs/pedigree worldwide, originated from well-known “founders” **(**Fig. 4a**, **Data_S1).

SBCMV response (both SEV and ELISA) appeared to be highly differentiated according to population structure, as shown in the box-plot phenotypic distributions based on population structure reported in Fig. 4b.

Germplasm of worldwide success as CIMMYT’60 (Jori C69 founder), CIMMYT’70 (Yavaros C79 founder), CIMMYT’80 (Altar C84 founder), the Italian (Valnova founder), and the North American and French germplasm (cvs. Kyle, Langdon, and Neodur, Vic, founders) are represented in the panel. CIMMYT’60, CIMMYT’70, and the North American/French groups showed a higher frequency of resistance as compared to other groups like the ICARDA temperate, while Italian varieties originated from Valnova and the CIMMYT’80 varieties originated from Altar C84 were all highly susceptible (Fig. 3). Additionally, resistance was observed in high frequency in old varieties derived from West Asian landraces (Haurani/Omrabi group, Fig. 3).

Population structure accounted for 21.5 and 13.3% of phenotypic variation for SEV and ELISA, respectively, indicating that SBCMV-resistant and SBCMV-susceptible alleles at *Sbm2* were unevenly partitioned among subpopulations/breeding groups. Figure 4b shows the NJ tree of the eight main subpopulations/breeding groups and the corresponding pattern of phenotypic distribution of SBCMV responses. Detailed population structure *Q* membership values together with phenotypic and genotypic data are reported in supplemental Data_S1 and Data_S2.

**Fig. 5:**
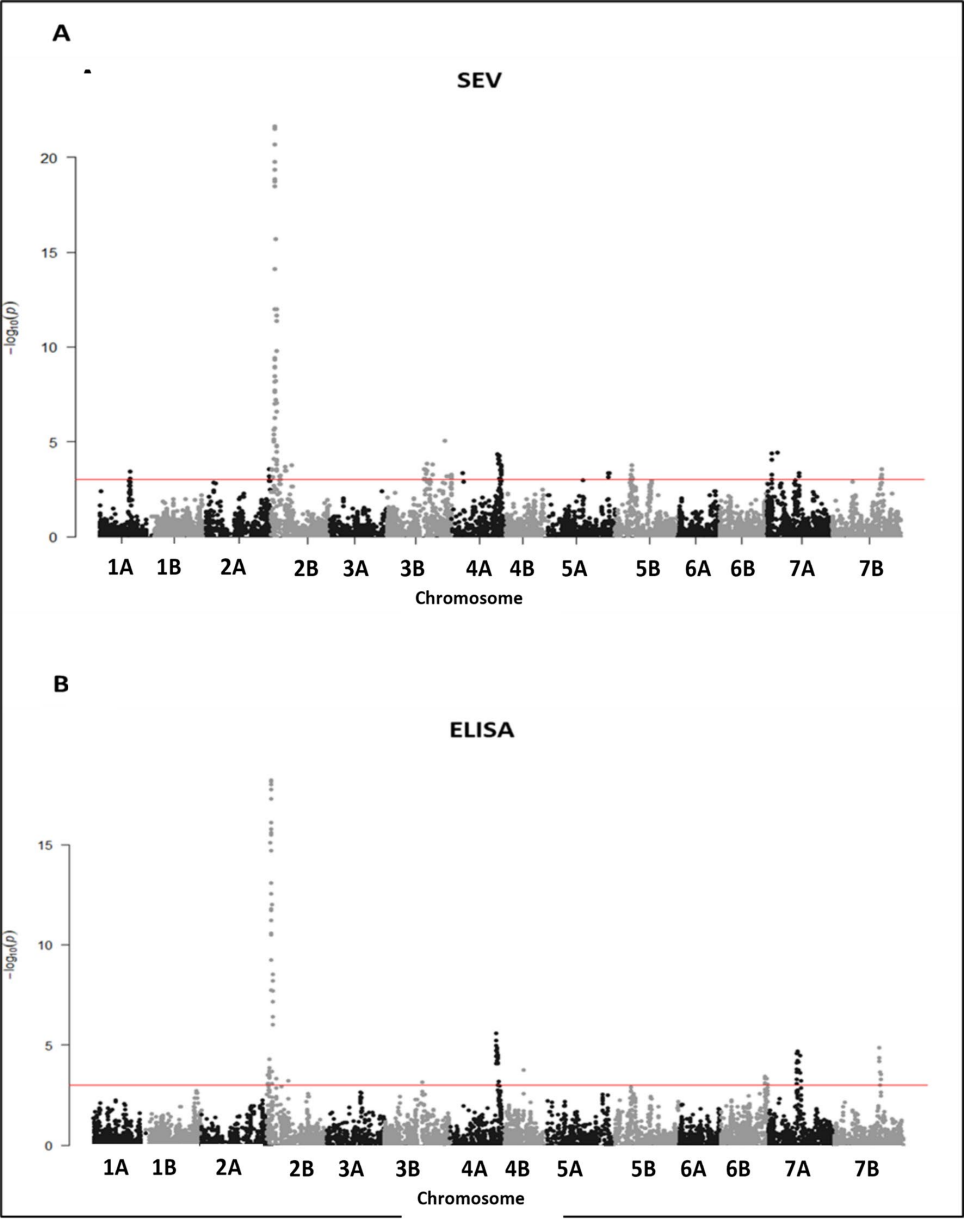
Manhattan plots of GWAS association analysis results for SBCMV response performed on the UNIBO Durum Panel. **A** GWAS for symptom severity scores (SEV; years 2005, 2007 and 2010). **B** GWAS for ELISA (years 2007 and 2010) data collected in Cadriano (Bologna, Italy). Red line represents the significance threshold set at -log_10_ (*P*-value) = 3

GWAS showed a major peak locus on chromosome 2B, corresponding to *QSbm.ubo-2BS* = *Sbm2* (Fig. 5). The LOD of this peak was 21.6 for visual score (SEV) and 18.2 for ELISA; *R*^2^ (= PVE) was 36.9% for visual score (SEV) and 34.1% for ELISA, respectively (Fig. 1). The three peak markers (*IWB72375*, *IWB67715,* and *IWB6584*) from the two SEV and ELISA phenotypes were coincident. Detailed marker–trait association results for the *Sbm2* region are reported in supplementary material section (Data_S2). Notably, the association results for SEV and ELISA were highly consistent.

The region corresponding to the peak of significance on Svevo genome extended from *IWB11421* (*KUBO*-27) at 13,011,332 Mb to *IWB6584* at 16,464,631 Mb, very close to the previously mapped *wPt-1601* at 16,497,383 Mb (Fig. 1 and Table 2). In this interval of ca. 3.5 Mb, 14 out of 35 markers showed very high LOD values, clearly identifying the *Sbm2* hotspot region. The entire region is tagged by the series *KUBO-27*, *wPt-2106*, *KUBO-29*, *KUBO-3*, *KUBO-41*, and *KUBO*-*40* (reported from distal -5′- to proximal -3′- 2BS chromosomal region, details of association results reported in Table 3), thus identifying the corresponding haplotypes highlighted in Fig. 3. Among KASP® markers (Table 3), the most closely associated is *KUBO-27*. Overall, − log_10_(*P*) ranged from 19.36 for *KUBO-27* from visual score (SEV) to 3.72 for *KUBO-13* as from ELISA test. The highest PVE was obtained for *KUBO-3* (36.4%, ELISA, Tables 2 and 3), while the lowest was obtained for *KUBO-13* (5.7%, ELISA; Tables 2 and 3) *Sbm2* allelic distribution appeared to be highly related to population structure, as all the founders of groups associated with resistance including ICARDA dryland, CIMMYT’60, CIMMYT’70, and North American / French varieties carried the *Sbm2*-resistant allele, while founders of ICARDA temperate, Italian, and CIMMYT’80 groups carried the *Sbm2*-susceptible allele (Data_S2).

**Table 2:** Peak markers in *Sbm2* chromosome region resulted from the GWAS analysis on the UNIBO Durum Panel

Marker	Position (cM)^a^	Position (bp)^b^	Strand ( ±)^c^	− log_10_ (*P*)	*R*^2^ (%)	Allele nucleotide	Allele frequency	Trait^d^
IWB72375	12.3	15,686,394	+	21.63	36.9	A/G	0.42/0.58	SEV_BLUEs
IWB67715	12.3	15,686,970	−	21.63	36.9	A/G	0.58/0.42	SEV_BLUEs
IWB6584	12.3	16,464,631	−	21.63	36.9	A/G	0.58/0.42	SEV_BLUEs
IWB72375	12.3	15,686,394	+	18.22	34.1	A/G	0.42/0.58	ELISA_BLUEs
IWB67715	12.3	15,686,970	−	18.22	34.1	A/G	0.58/0.42	ELISA_BLUEs
IWB6584	12.3	16,464,631	−	18.22	34.1	A/G	0.58/0.42	ELISA_BLUEs

^a^Genetic map positions (cM) are reported as in the consensus map of Maccaferri et al. (2015)

^b^Physical map positions (bp) are reported as the start positions obtained by blasting the marker sequence on the durum wheat genome reference Svevo (Maccaferri et al. 2019)

^c^Strand defines based on the direction of the marker sequence

^d^Best linear unbiased estimates (BLUEs) were calculated based on LUEs of A) symptom severity scores (SEV) data from 2005, 2007, and 2010 (SEV_ BLUEs) and of ELISA data collected in 2007 and 2010 (ELISA_BLUES) in Cadriano (Bologna, Italy), spatially corrected

**Table 3:** Association results for KUBO KASP® markers and *wPt-2106* in GWAS performed on the UNIBO Durum Panel

Marker	Position (cM)^a^	Position (bp)^bs^	Strand ( ±)^c^	Resistant allele	Susceptible allele	Allele frequency	SEV^d^		ELISA^d^	
							− log_10_(*P*)	*R*^2^ (%)	−log_10_ (*P*)	*R*^2^ (%)
KUBO-13	8.4	9,986,862	+	A	G	0.45/0.45	5.65	8.3	3.72	5.70
KUBO-27	11.6	13,011,332	+	A	G	0.56/0.39	19.36	35	17.76	35.6
wPt-2106	10.9	14,314,449	−	1	0	0.38/0.59	18.48	31.2	15.1	27.8
KUBO-29	11.6	15,643,691	+	G	T	0.57/0.38	18.84	32.4	16.12	30.9
KUBO-1	12.2	15,672,582	−	T	C	0.56/0.26	14.11	34	15.49	35.9
KUBO-3	12.3	15,805,908	+	T	C	0.26/0.57	11.99	26	15.78	36.4
KUBO-41	12.3	16,184,452	−	C	T	0.68/0.25	6.27	9.3	10.58	19.6
KUBO-40	12.3	16,185,445	+	C	T	0.58/0.25	9.35	19.2	11.75	25.6
KUBO-9	19.0	24,313,744	+	G	A	0.26/0.66	9.8	16.9	7.17	12.6

^a^Genetic positions (cM) are reported as in the consensus map of Maccaferri et al. (2015)

^b^Physical positions (bp) are reported as the start positions obtained by blasting the marker sequence on the durum wheat genome reference Svevo (Maccaferri et al. 2019)

^c^Strand defines the direction of the marker sequence

^d^Best linear unbiased estimates (BLUEs) were calculated based on symptom severity scores (SEV) data from 2005, 2007 and 2010 (SEV_BLUEs) and ELISA data collected in 2007 and 2010 (ELISA_BLUES) in Cadriano (Bologna, Italy), spatially corrected

Several minor putative QTLs across the genome resulted from the GWAS (Table S4). Up to 20 putative minor QTLs at − log_10_(*P*) > 3 in GWAS were mapped, eight of which were common to both SEV and ELISA (Tables S4a and S4b, for SEV and ELISA, respectively).

Minor QTLs common to both SEV and ELISA were found on chromosomes 2A (*IWB71381*, 206.5 cM), 2B (two QTLs, at *IWB6584*, 12.3 cM, and *IWB48240,* 67.6 cM), 4A (two QTLs, at IWB6276, 147.4 cM and *IWB37657*, 156.9 cM), 5B (*IWB67284*, 53.8 cM), 6B (*wpt*-*9589*, 153.8 cM), 7A (*IWB72581*, 102.4 cM), and 7B (*IWB71789*, 146.2 cM).

After completing the GWAS, the *QSbm.ubo-2BS* = *Sbm2*, the additional minor QTLs, and population structure Q values were modeled in GLM (Table S5). Major and minor QTL and population structure effects on SEV and ELISA were tested based on a multi-marker general linear model (*MM-GLM* + *Q*). The *p*-values and adjusted *R*^2^ of the full *Sbm2* and minor QTL models are reported in Table S5**.** The population structure *Q* memberships accounted for 21.5 and 13.3% of SEV and ELISA blues values, respectively, a finding mainly explained by the allele distribution at *Sbm2*, whose resistant and susceptible alleles were differently distributed according to main population structure partitioning. Therefore, we modeled the effect of *Sbm2* together with the additional minor QTLs suggested by GWAS in a GLM excluding population structure as covariate**.** Notably, *Sbm2* accounted for 54.6 and 46.9% of the total phenotypic variation for SEV and ELISA, respectively. Minor QTLs were mostly confirmed by GLM for either SEV or ELISA, or both, except for *IWB48240*_*2B* and *IWB26062*_*4A*, which were not validated by the GLM. *Sbm2* × other QTL interactions were mostly non-significant. Importantly, the addition of minor QTL effects and their interactions raised the total variation explained by the global genetic effects up to 67.6 and 54.7% for SEV and ELISA, respectively **(**Table S5**).**

The distribution of resistant and susceptible alleles at *Sbm2* and minor QTLs in the UNIBO durum panel is detailed in Data_S3. Resistant alleles at minor QTLs were distributed across multiple varieties, from a minimum of four to a maximum of 18 alleles per variety. The linear model plot reports the cumulative number of resistant alleles across different genotypes in the X-axis, based on the tag-QTL marker scores, whereas the Y-axis reports the BLUES of SEV and ELISA data. Based on this data, linear regression was computed to estimate the overall effect of minor QTL combinations on the phenotypic data (Data_S3).

The global effect of the susceptible-to-resistant ideal progressive substitution of alleles in germplasm is exemplified by the histograms reported in Data_S3, reported separately for varieties carrying either the resistant or susceptible allele at *Sbm2*.

In the 148 *Sbm2* resistant varieties, the cumulated substitution effect of minor QTLs was estimated (based on the regression reported in Data_S3) to improve SEV only marginally from 1.63 (four minor resistant alleles) to 0.92. However, the effect was more marked on the ELISA value that decreased from 0.92 (4 minor alleles) to 0.40 (18 alleles).

Minor QTL allelic substitution effect impacted more evidently on the group of 110 *Sbm2* susceptible varieties, as expected. In this group, the SEV response of varieties decreased from 3.81 (varieties cumulating four minor resistant alleles only) to 1.82 (varieties cumulating 16 cumulated minor resistant alleles) and, for ELISA, the average value decreased from 1.45 (four resistant alleles) to 0.94 (16 resistant alleles), thus providing a substantial improvement in resistance.

### Fine mapping of *Sbm2* based on the Svevo × Ciccio and Meridiano × Claudio RIL populations

Genetic maps constructed for each population are presented in Fig. S4**.** KASP® order was coincident in the two maps. In the Mr × Cl map, *KUBO-27* was in a recombination bin including *KUBO-29* and *wPt-2106*, while in the Sv × Cc map it was separated by a recombination event. KASP® and *wPt-2106* genotypes of the Sv × Cc RILs are reported in Table S5a. Crossing overs were not evenly distributed in the target interval. Only seven RILs showed recombination inside the target interval and were therefore informative for fine mapping (Table S5b). In total, 433 recombinant Sv × Cc RILs in the *KUBO-13* to *KUBO-9* interval were selected and scored for SBCMV resistance in 2016 and 2017. BLUEs frequency distribution obtained from corrected SEV data is reported in Figure S6. Data presented a mean value of 2.58 with extreme values of 0.29 and 4.27. The distribution histogram showed two peaks, one centered on a SEV score of 1.53 (medium resistant) and one on a SEV score of 3.16 (medium susceptible), with heritability value of 0.83.

The joint genotypic and phenotypic analysis of the Sv × Cc recombinant lines allowed exclusion of the two distal regions marked by *KUBO-13* and *KUBO-9* flanking markers. Sixty-five resistant lines showed that the QTL was most probably downstream of *KUBO-13* and 140 susceptible lines showed that it was upstream of *KUBO-9* (Table S6a). Three recombinant susceptible lines (#652, #2077, #3089) together with three recombinant resistant lines (#1450, #126, #1166) showed that the QTL is most probably upstream of *KUBO-1* located at 13,011,332 bp in Svevo (Table S6a). However, only one additional recombinant line (#139) provided evidence for the QTL to be downstream of *KUBO-27* (Table S6a). Therefore, Sv × Cc RILs positioned the supporting interval of *Sbm2* in 0.2 cM between *KUBO-27* and *KUBO-1,* corresponding to 2.661 Mb.

**Fig. 6:**
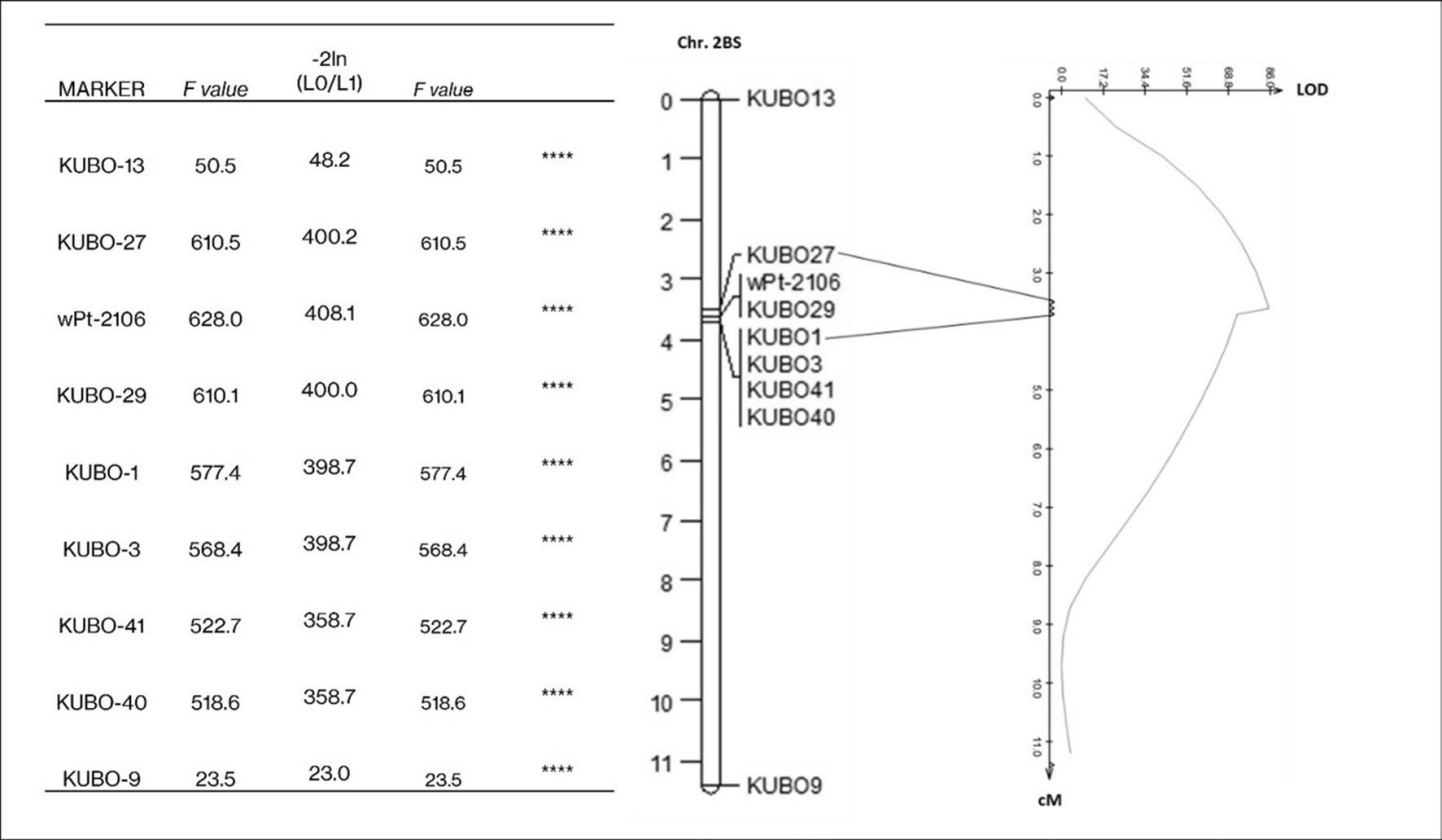
Single-marker QTL analysis results for *KUBO* KASP® markers and *wPt-2106* mapped in Sv x Cc population. Interval mapping was based on SEV trait data collected in 2016 (3 dates) and 2017 (5 dates) in Cadriano (Bologna, Italy) for *Sv x Cc* RILs

As to the Mr × Cl RIL population, beside the confirmation of results from the Sv × Cc populations, 18 RILs were informative for fine mapping **(**Table S6b**)** and confirmed the interval downstream of *KUBO-13* (supported by five RILs) and upstream of *KUBO-1* (supported by two recombinant lines) as the most probable for harboring *Sbm2*. Single-marker analysis (Fig. 6) showed that *wPt-2106* was the marker most associated with the phenotype. Consistently with GWAS, *KUBO-27* was the most associated with the phenotype among all newly developed KASP® markers. The result of the Interval Mapping analysis is reported in Fig. 6. As expected, the LOD value peaked in the region identified by *KUBO-27, wPt-2106, KUBO-29,* and *KUBO-1*, with a peak LOD value of 86.

### Comparison among genomes for marker and gene order in *Sbm2* region

A comparison of marker order in *Sbm2* region among the three wheat genomes (cvs. Zavitan, Svevo, and Chinese Spring) is shown in Figure S6. Order inconsistency among physical maps of the three genomes was observed for 26 markers, mostly located in a few regions that showed rearrangements among assemblies, as in the case of a group of eight consecutive markers (*IWB71947, IWB35524, IWB72156, IWB62546, IWB41644, IWB73884, IWB29097,* and *IWB72157*) that identified an inversion of ca. 2 kb in Svevo**.** The three wheat genomes were therefore compared for gene collinearity in the interval *IWB73347* (*KUBO-13*, position: 9,986,862 bp) and *IWB10512* (*KUBO-9*, position: 24,313,794 bp), including *Sbm2*. This analysis further confirmed the local inversion spanning the region tagged by *IWB45885, IWB4596, IWA7545, IWB26871, IWB26232, IWA3589,* and *IWB26233* (Fig. S7). No inversions were detected between Zavitan and Chinese Spring assemblies, indicating that the local inversions in Svevo genome could be an artifact of assembly. Additional analysis using long-read sequencing data is required to confirm this observation and its potential effects on differential gene expression.

### *Sbm2* molecular haplotypes in cultivated durum germplasm

With the aim to define the linkage disequilibrium and haplotype patterns in the *Sbm2* region, SNP data from the UNIBO durum panel were subjected to local disequilibrium analysis using the linkage block method implemented in the Haploview software. Considering the extended region between *KUBO-13* and *KUBO-9*, six main linkage blocks of various Mb size were identified (Fig. 2). The target interval on chromosome 2B is comprised between 9,986,912 and 24,313,794 bp. Based on the block partitioning, both fine mapping and GWAS results indicate that *hapblock-3* harbors the QTL hotspot*.*

SNPs were partitioned into six haplotype blocks (*hapblocks*) as in Fig. 2 and their − log_10_ (*P*) of association with SBCMV response are reported as horizontal heat bars in the lower side of the figure (F-G panes).

Detailed local SNP-based haplotypes present in the UNIBO Durum Panel varieties for the *KUBO-13* to *KUBO-9* region are reported in Fig. 3 and in Data_S2. In Fig. 3, varieties were clustered based on the identity-by-state (IBS) genetic distance matrix and Ward’s clustering obtained using SNPs from the *Sbm2* region only (Fig. 3, A pane), thus reflecting the genetic relationships among varieties based on *Sbm2* haplotypes only. In Fig. 3, clustered varietal haplotypes are shown side by side with their respective whole-genome population structure *Q* values (eight* Q* subpopulations, reported as heat bar), SEV- and ELISA-BLUES values, all reported as vertical heat bars aligned from left to right (Fig. 3, B and C panes). Haplotypes of varieties/breeding lines at *Sbm2* are shown as horizontal bars adopting a two-color scheme where the SNP allele from the resistant Meridiano, Levante, and Neodur parents was considered as the main reference allele and hence is dark blue colored, while the alternative SNP allele, carried by susceptible parents, is pale red colored (Fig. 3, D, E, and F panes).

Considering the region as a whole, two main haplotype groups (H1 and H2) were identified and were clearly associated with resistance (H1-R) and susceptibility (H2-S and H3-S), respectively. Within resistant and susceptible haplotype groups, sub-haplotypes were defined based on haplotype differentiation, termination, and/or recombination events among haplotypes (indicated as black arrows, numbered from 1 to 6). Additional haplotypes were observed in a group of a few landraces only with a much more complex haplotype structure, mostly associated with a susceptible response (bottom side of the figure). R- and S-haplotype groups showed population structure effects, with H1-R being more frequent in North American, French, CIMMYT’70, Desert Durum, and some ICARDA temperate varieties. Conversely, H2-S was more represented in ICARDA dryland, Italian, French, and Spanish varieties derivatives of Valnova/Simeto while H3-S was specifically found in highly susceptible varieties derived from the CIMMYT’80 Altar84 founder.

SNPs differentiating the H1-R group from the two H2-S and H3-S groups can already be identified, from left to right, in *hapblock-1* (*IWB73347* = *KUBO13*, A/G, indicated by black arrow 1) and *hapblock-2* (including all SNPs of the block, reported in Data_S2**)**. However, these SNPs are highly recombined with the phenotype and have low discrimination power, indicating that *hapblock-1* and *-2* are distant from the causal sequence. For instance, at *IWB73347* = *KUBO-13*, the G variant (in pale red) is present in relatively high frequency in both R and S varieties. Similarly, all SNPs included in *hapblock-2* showed variants common to both R and S varieties and were therefore unable to discriminate between resistant and susceptible varieties, while the rare variants discriminated between modern and ancient germplasm only.

*Hapblock-3* starting in correspondence of black arrow 2 in Fig. 3 (*IWB11421* = *KUBO-27*) and ending with SNP *IWB23330* showed the highest density of SNPs with the highest association to SBCMV response and the highest resolving power for H1-R and H2-S haplotypes (*IWB11421* = *KUBO-27, IWB42660, IWB12133, wPt-2106, IWB50507, IWB40895, IWB23029* = *KUBO-29, IWB72375, IWB67715, IWB73884, IWB71947, and IWB6584*), three of which were transformed into breeder friendly markers suitable for marker-assisted selection (*KUBO-27*, *wPt-2106, and KUBO-29)*.

Within *hapblock-3*, a main H1-R haplotype associated with resistance and two haplotypes associated with susceptibility, H2-S and H3-S, can be clearly distinguished. Interestingly, they showed a core haplotype region common to both S haplotypes and highly differentiated from H1-R*,* comprised between *IWB11421* = *KUBO*-27 (black arrow 2 in Fig. 3) and *IWB28973* = *KUBO-1* (black arrow 3). From *KUBO-1* downwards, still in *hapblock-3*, the two S haplotypes start to differentiate as to each other and differentiate from H1-R for a wide segment. Therefore, based on GWAS results, the *KUBO-27* to *KUBO-1* interval is the main candidate to harbor the *Sbm2* causal sequence.

**Fig. 7:**
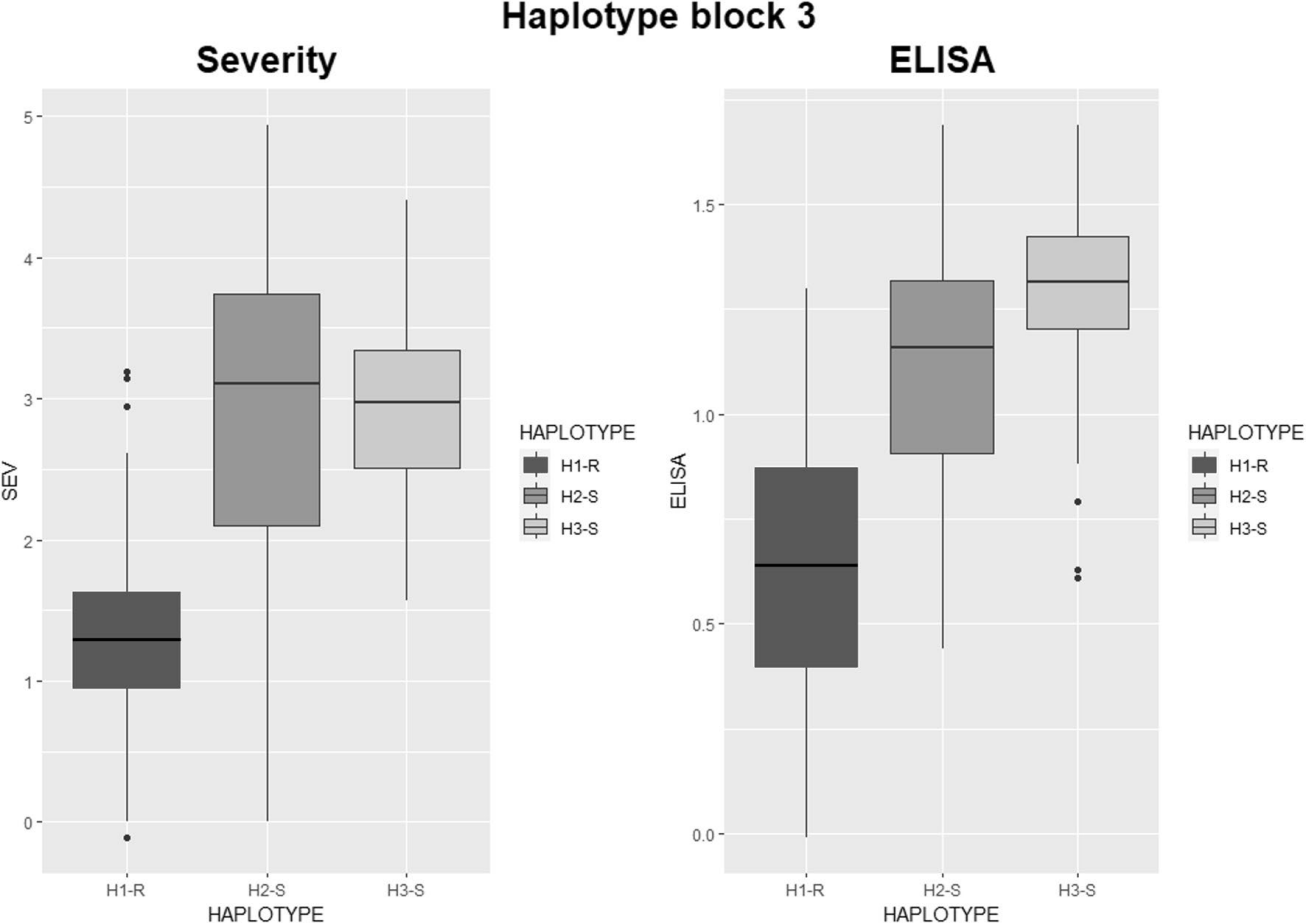
Distribution of phenotypic values (y-axis) for UNIBO Durum Panel varieties and breeding lines, based on the main three haplotypes detected in *hapblock3* (x-axis) for SEV (panel **A**) and ELISA (panel **B**) phenotypic values (BLUEs). Phenotypic data were collected in 2005 (SEV), 2007 (SEV and ELISA) and 2010 (SEV and ELISA) in Cadriano (Bologna, Italy) and spatially corrected. Haplotype one is associated with resistance (H1-R) and haplotype two (H2-S) and three (H3-S) are associated with susceptibility

On the proximal side, *hapblock-4*, *-5,* and *-6* all showed SNPs weakly associated with SBCMV, with resolving power considerably lower than those in *hapblock-3*, clearly indicating *hapblock-3* as the most relevant region for *Sbm2.* Interestingly, H1-R haplotype included a group of resistant cultivars with sub-haplotype H1c-R (mainly ICARDA dryland varieties) showing a haplotype clearly recombined with H2-S particularly in *hapblock-5* and *-6.* Overall, these results indicated *hapblock-3,* harboring the QTL hotspot. The phenotypic effect on SEV and ELISA of the three main haplotypes present in *hapblock-3* and tagged by the multi-marker *KUBO-27, wPt-2106, KUBO-29, KUBO-1, KUBO-3, KUBO-41,* and *KUBO-40* series is reported as box-plot in Fig. 7**.**

Overall, these observations are consistent with the results obtained from the fine mapping experiment with the bi-parental populations.

### Haplotype distribution in a panel of worldwide durum wheat varieties across decades

To broaden the survey regarding *Sbm2* haplotypes in cultivated durum wheat, a larger panel of 549 varieties was investigated and genotyped with diagnostic markers *KUBO-27, KUBO-29,* and *KUBO-1*. Based on the genotyping results, haplotypes were classified in categories: resistant haplotype, susceptible haplotype, recombinant haplotype of type-1, type-2, type-3, double recombinant haplotype, and admixed. The proportion of accessions represented in each haplotype category is reported in Figure S8. The resistant haplotype was the most frequent, being detected in 53% of the accessions, followed by the susceptible haplotype detected in 31% of accessions. The three categories of recombinant haplotype type-1, type-2, and type-3 represented in total 11%, while accessions with a double recombinant haplotype were less than 1%.

**Fig. 8:**
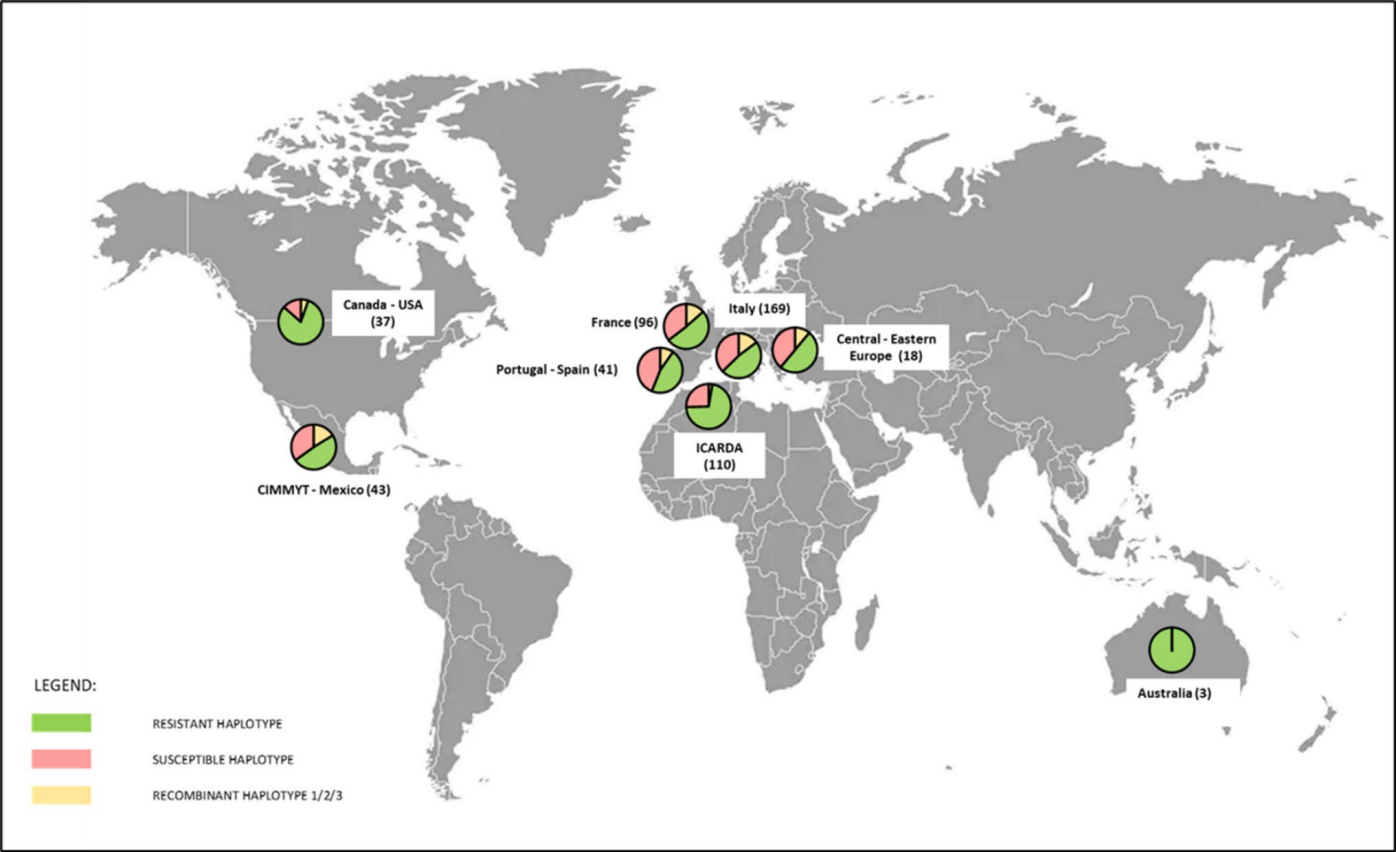
Proportion of SBCMV-resistant, SBCMV-susceptible, and recombinant haplotypes in durum wheat varieties based on their origin. The number in brackets refers to the total number of accessions. The haplotype refers to three markers (*KUBO-27*, *KUBO-29* and *KUBO-1*) and includes the *Sbm2* support interval

The distribution of resistant, susceptible, and recombinant haplotypes based on varietal origin is reported in Fig. 8. For all origins, varieties with the resistant haplotype represented more than 45% of the total, with Portugal–Spain having the minor proportion of resistant accessions (46%). The highest proportion of resistant varieties was observed for the Canada–USA (81% resistant varieties) and the lowest for CIMMYT–Mexico (49% resistant varieties). In Europe, the resistant haplotype was mostly represented in France (51%), even if the proportion was very similar to that observed for the other European countries, ranging from 46 to 51%. ICARDA materials showed the presence of the resistant haplotype in relatively high frequency (72%). The susceptible haplotype was poorly represented in Canada–USA (14%) and ICARDA (25%), while ranged from 35 to 44% for all the other locations.

Time course haplotype distribution trends were inspected from 1955 to 2020 (Figure S9). Notably, the registration of resistant varieties in North America underwent an outburst in the 1995–2000 interval. Additionally, in Italy and France the release of varieties with the resistant haplotype increased starting from 2000. As to Portugal–Spain, the release of varieties is more evenly distributed throughout the years, with a major release of resistant varieties in the interval 1995–2000. Release of varieties from ICARDA was concentrated in the first half of the considered period, from 1985 to 2000, and most of the novel varieties presented the resistant haplotype (Fig. 8).

### Candidate gene analysis at *Sbm2*

**Fig. 9:**
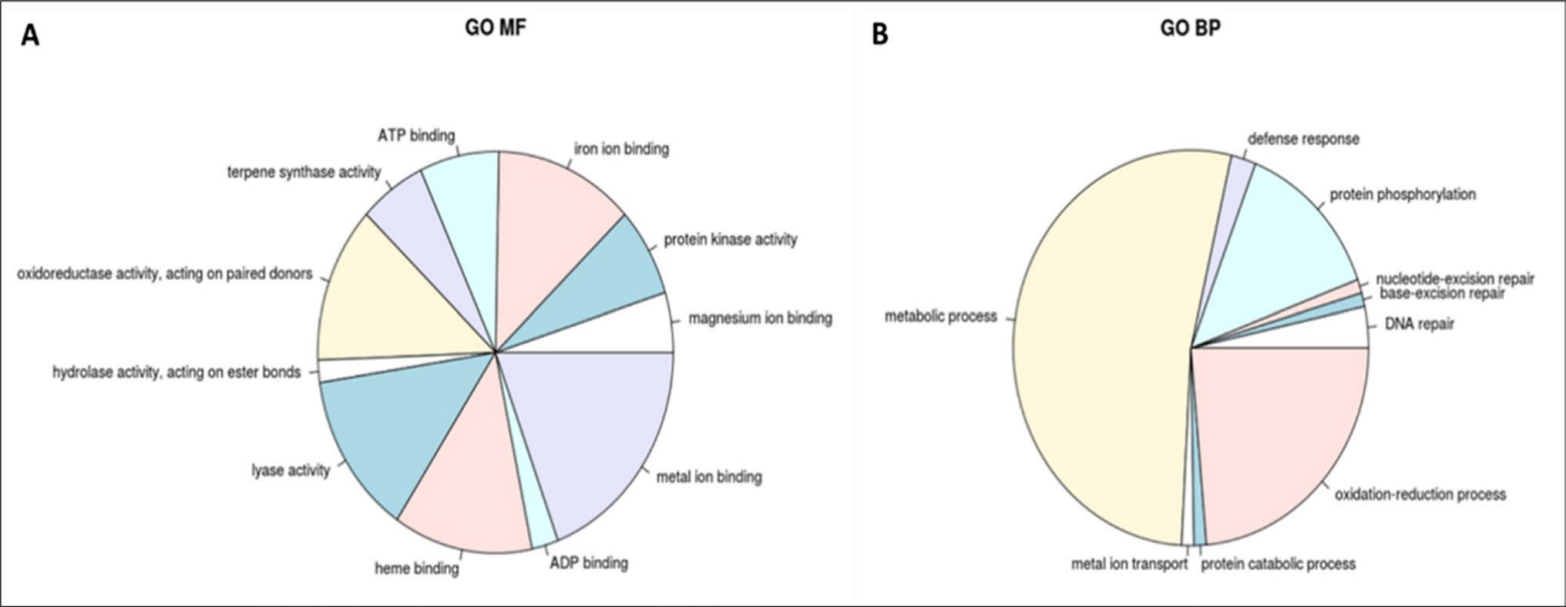
Molecular functions (**A**) and biological processes (**B**) of genes present in the support interval of *Sbm2* based on Gene Ontology

The *hapblock-3* hotspot interval between *KUBO-27* and *KUBO-1* up to *KUBO-40/IWB6584/wPt-1601* corresponded to 3.2 Mb on the Svevo genome (Maccaferri et al. 2019), a region harboring a total of 93 genes (Table S8), of which 52 were high-confidence (HC) and 41 low-confidence (LC) genes. Genes present in this region appeared to be involved mainly in metabolic, oxidation–reduction and protein phosphorylation processes (Fig. 9). Part of those genes are involved in plant defense response, hence representing candidate genes putatively involved in SBCMV resistance. Such genes belong to defensins, disease resistance protein (NBS-LRR class) family and NBS-LRR-like resistance proteins (Table S7). In the fine-mapped interval, we observed: 17 cytochrome p450, three receptor kinases, two defensins, one high-confidence and one low-confidence, and three NBS-LRRs in addition to further genes involved in secondary metabolic pathways and transporter activity. Defensins class included an HC gene and a LC gene. As regards to the NBS-LRR genes (HC), two of them encoded for NBS-LRR resistance proteins, while one was considered an NBS-LRR like resistance protein.

Based on the gene ontology enrichment (GO), the molecular functions ascribed to mono-oxygenase activity, terpene biosynthesis, and ion binding are highly represented in the *Sbm2* confidence interval between markers *KUBO-27* and *KUBO-40,* based on the logarithm of adjusted *p*-value (Fig. 9)*.* With regard to the biological processes, general metabolic pathways are highly frequent such as terpenoid and isoprenoid biosynthetic processes and protein phosphorylation (Figure S10), classes known to be involved in the plant–pathogen interaction.

The genes included in the interval between *KUBO-27* and *KUBO-40* were also explored for KEGG terms using *BlastKoala* bioinformatic tool (Kanehisa et al. 2016). As to the organism system attribution, the main represented pathway is "plant–pathogen interaction," which reports the metabolic pathways involved in the recognition between a host organism and a virulent pathogen. As shown in Fig. S11, the gene *NBS-LRR RPM1* (*TRITD2Bv1G007240*) appears to be involved in this pathway as responsible for the hypersensitive response (defense response) as a consequence of the recognition between the NBS-LRR RPM1 product and the pathogen avirulent protein (avr).

Additionally, the gene content in the *Sbm2* confidence interval was compared to the candidate genes identified in the fine-mapped *Sbm1* region on chromosome 5DL of bread wheat by Liu et al. (2020). The *Sbm1* interval was positioned between 546,651,779 bp and 547,273,461 bp, harboring 17 candidate genes from CS RefSeq v1.0 pseudomolecule (IWGSC, 2018). The candidate gene functions belong to Ser/Thr kinase proteins, phosphorylation proteins for signal transduction, membrane transporter binding proteins, transcription factor proteins, and secondary metabolism proteins. Functions such as secondary metabolism, signal transduction by protein phosphorylation, and in particular Ser/Thr proteins and transcription factors are shared with gene functions reported for *Sbm2* (Table S8). However, Liu and colleagues (2020) identified as the most promising candidate a pto-interacting protein 1 (PTI1) (*TraesCS5D01G531200*) based on RNA-Seq experimental data. This specific gene function was not identified among the *Sbm2* candidate genes which, on the other hand, represented other promising candidates. With the aim to find ortholog and/or paralog genes shared between *Sbm1* and *Sbm2* durum and bread wheat intervals, the ENSEMBL plant database where orthologs and paralogs of each annotated gene are listed based on alignment identity, common domains, and molecular function was surveyed. The comparison between *Sbm2* and bread wheat CS RefSeq v1.0 genome was also performed, retrieving orthologs in CS reference genome and paralogs in Svevo durum wheat genome. The results of the ENSEMBLE search are reported in Data_S4 and Data_S5. As to *Sbm1* 5D genes, no orthologs/paralogs were identified in the *Sbm2* region of chromosome 2B. Most of the *Sbm1* 5D orthologs mapped to 4A and 5B chromosomes, as expected based on the wheat 4AL/5AL translocation (Devos et al. 1995), and not on chromosome 2 group. On the other hand, mapping of *Sbm2* orthologs and paralogs, showed absence of the corresponding orthologs/paralogs in *Sbm1* 5D in Chinese Spring and Svevo genomes.

## Discussion

### Fine mapping of *Sbm2*

This work was based on the previous knowledge gathered on the genetic basis of SBCMV resistance in durum wheat and was aimed at fine mapping the unique major QTL responsible for this mechanism known so far in durum (Maccaferri et al. 2012). The fine mapping strategy took advantage of the combination of both production of novel recombinants obtained by artificial crossing and use of GWAS in a panel of varieties cultivated worldwide.

Based on these results, the most informative Illumina SNPs mapped in the tetraploid wheat consensus map and in the Svevo genome assembly (Maccaferri et al. 2015 and 2019) were successfully converted to KASP® assays paving the way for haplotype tracing, germplasm characterization, and MAS (Koebner and Summers 2003; Maccaferri et al. 2022). SNP markers proved very effective for MAS when coupled with a high-throughput technology which allows greater flexibility as compared to the original SNP array technology (Shavrukov 2016). The 69% SNP-to-KASP conversion rate allowed for an effective exploitation of SNP markers singled out with the Illumina SNP array. The probability of success in converting an SNP to a KASP® marker, however, varied greatly according to each SNP. For some SNPs, the design of suitable codominant KASP® assays was particularly difficult or impossible, as already reported by Makhoul et al. (2020).

To fine map *Sbm2*, a combination of GWAS and linkage mapping in progenies derived from two different populations, namely the Sv × Cc and the Mr × Cl RILs, was used (Salvi and Tuberosa 2005). Recombinants were identified with two flanking KASP® in a 2.0 cM interval. RIL observations confirmed what was reported by Maccaferri et al. (2011a; 2012) on *Sbm2* as a major locus for SBCMV resistance in durum wheat. The divergence of the phenotypic response from a normal distribution toward a bimodal distribution supports this hypothesis. However, the absence of a clear bimodality suggests the influence of additional QTLs with minor effects, an expected outcome of QTLome dissection (Salvi and Tuberosa 2015).

The spatially adjusted model applied to phenotypic data analysis effectively allowed for accounting micro-environmental variation and uneven incidence of SBCMV infection across the field, scoring/sampling date, and seasons. It is well known that SBCMV infection effects are strongly influenced by environmental factors such as temperatures and soil humidity that impact both vector and virus spreading, hence causing spatial patchiness of infection incidence (Bayles et al. 2007; Liu et al. 2014). Moreover, effects of cold weather on plant growth can worsen virus symptoms. For these reasons, implementing growth chamber experiments under controlled conditions is highly desirable to standardize infection effects and to increase heritability.

The frequency of informative recombination events between *KUBO-13* and *KUBO-9* obtained from the two mapping populations was highly heterogeneous and considerably lower in Sv × Cc as compared to Mr × Cl. This was possibly due to local cross-specific suppression of recombination (Akhunov et al. 2003; Conley et al. 2004), supporting the need for diverse genetic materials. Maccaferri et al. (2019) reported that the chr. 2BS telomere of the Svevo genome is highly recombinogenic, with an average physical-to-genetic ratio of 1.8 Mb/cM. For the interval between *KUBO-13* and *KUBO-9,* our results demonstrated a physical-to-genetic ratio of 1.3 and 2.5 Mb/cM for Sv × Cc and Mr × Cl RILs, respectively. However, in correspondence of the fine-mapped interval of *QSbm.ubo-2BS* interval between *KUBO-27* and *KUBO-1*, the ratio was 3.1 Mb/cM for Mr × Cl and 13.5 Mb/cM for Sv × Cc*.*

The complementary GWAS analysis conducted on modern cultivars from around the world allowed mapping *Sbm2* to the same short interval obtained from the RIL fine mapping experiments, hence providing an independent validation of results, while enabling the evaluation of allelic distribution and diagnostic power of significant SNPs in cultivated germplasm. All markers were validated and the assessment of their diagnostic power highlighted *wPt-2106_ASO, wPt-2106_HRM, KUBO-27, KUBO-29, KUBO-41,* and *KUBO-3* as the best markers for MAS.

The potential to predict the phenotype at the causal locus among genotypes belonging to different breeding origins/lineages is important for wide application of MAS in diverse genetic backgrounds and contributes to define the breeding value of a marker (Terracciano et al. 2013). A drawback of SNP markers, as compared to SSR markers, is their biallelism, as SNPs fit with the infinite site and allele mutation model (Kimura and Crow 1964; Kimura 1969). This strongly limits the chance of a single SNP marker to consistently match with the causative mutations and haplotypes at the QTL. Notably, the availability of multiple SNP markers tightly associated with the target locus allows for more accurate haplotype-based screening, a more effective approach for increasing the diagnostic power of MAS (Meuwissen et al. 2001; Mucha and Wierzbicki 2012) and a more effective breeding by design approach (Peleman and Voort 2003).

In this study, several concatenated SNPs highly diagnostic of the allelic state at *Sbm2* have been identified and some of them were successfully transformed into high-throughput KASP® or HRM markers, in sufficient number to perform haplotype-based characterization. This enables to trace identity by descent of *Sbm2 alleles* at high level of confidence. The combination of *KUBO-27, wPt-2106, KUBO-29*, *and KUBO-41* provides the best multi-marker haplotype assay available to ascertain the presence of the resistant-*Sbm2* allele for germplasm characterization, including diverse breeder’s materials, segregating individuals, and lines from artificial crosses, while characterizing selected breeding lines as possible candidate for varietal registration.

Until now, *Sbm2* has been considered the only major QTL to provide SBCMV resistance in durum wheat (Bayles et al. 2007; Maccaferri et al. 2012). If additional major sources of resistance are found in *Triticum turgidum* and/or other tetraploid wheat germplasm, or in chromosomes A and/or B of bread wheat, it would be useful to pyramid them into new varieties to increase both virus resistance and durability; hence, the KASP® markers developed in this study are instrumental.

Recently, the *Jmv1* locus for resistance against the *Japanese soil-borne wheat mosaic virus*, a Furovirus also transmitted by *Polymyxa graminis*, has been identified in chromosome 2H of barley (Okada et al. 2020 and 2023). A joint comparative analysis of the two loci in Triticeae would increase the chance to better understand the causal locus and mechanism of resistance.

### Candidate genes in *Sbm2* interval

The Svevo genome enabled the identification of the gene(s) responsible for *Cdu-B1,* the major locus underlying the cadmium concentration QTLome in grain, mainly based on candidate gene analysis and translational genomics, previously hindered by the low recombination rate in the target region (Maccaferri et al. 2019). A similar approach could be used to dissect the *Sbm2* region. Other solutions are represented by alternative cloning approaches which do not rely on positional fine mapping, like the MutRenSeq method proposed by Steuernagel et al. (2016).

A glance at the list of candidate genes proposed in this study identified three gene categories as most promising for underlying SBCMV resistance: defensins, disease resistance protein (NBS-LRR class) family, and NBS-LRR-like resistance proteins. Plant defensins are a family of folded antimicrobial peptides thought to be effective against fungal pathogen (Thomma et al. 2002), while NBS-LRR resistance proteins represent the largest class of plant resistance genes (*R* genes) and *R* genes are involved in resistance against diverse pathogens, including viruses (Calil and Fontes 2017).

Specifically to SBCMV, Vallega et al. (2006) proposed a hypothesis about a generalized resistance to virus accumulation in durum wheat, i.e., a mechanism controlling virion accumulation in plants by limiting the spread of the virus particles from the roots to the shoot. Kanyuka et al. (2004), Ordon et al. (2009), and Perovic et al. (2009) reported a potential *Sbm1* mechanism defined as “translocation resistance,” which prevents the virus to spread from the roots to the stem and leaves in SBCMV-resistant hexaploid cultivars. However, a more complete explanation about the mechanism of SBCMV defense in wheat has not yet been sufficiently defined. Thus, it would be inappropriate to narrow down the search of candidate genes to the *R* gene category only. Singh and Sharma (2015) reported that several terpenoids have a role in plant defense, acting as phytoalexins, low molecular weight compounds, known to exhibit antimicrobial properties in rice (Prisic et al. 2004) and deterrent for insects and herbivores in corn, lima bean, poplar, and cotton (Rodriguez-Saona and Crafts-Brandner 2003; Arimura et al. 2004; Mithofer 2005; Schnee et al. 2006). Ten genes involved in terpene synthase activity were detected in the *Sbm2* region, representing the second most frequent gene category. Another uncertainty lies in disentangling the direct resistance against the virus from the indirect resistance against the *Polymyxa* vector, particularly because *P. graminis* is not well known as *Polymyxa betae*. Additionally, with the objective of providing an overall view of the entire list of genes present in the confidence interval, we did not exclude low-confidence genes. High-/low-confidence classification based on a single reference genome is not a valid criterion to discriminate putative candidate genes. Jupe et al. (2012), in a survey of NBS-LRR genes in the potato genome, showed that many of these genes previously annotated as partial/low confidence by the Consortium (2011) were actually functional.

Herein, an overall view on the list of genes presents in the confidence interval of the Svevo durum wheat reference genome and resistance donor was outlined, providing a starting point for a deeper and detailed scan of functions and biological processes.

## Conclusions

In this study, SNP markers were used to dissect the genetic basis of SBCMV resistance in durum wheat. High-density SNP arrays allowed for marker enrichment of *QSbm.ubo-2BS* = *Sbm2* region, while simple, cheap, and robust SNP-specific assays are required for targeted high-throughput screening such as fast evaluation of large segregant populations and efficient marker-assisted selection. Among the available technical solutions, KASP® technology is currently the most used worldwide. Fine mapping of *Sbm2* was achieved, with *Sbm2* now delimited to a 0.2-cM interval including *wPt-2106* and flanked by *KUBO-27* and *KUBO-40-KUBO-41*, overlapping the target QTL.

This study provides a series of markers organized into haplotypes closely associated and overlapping to the target QTL, hence highly diagnostic for resistance. Such markers are instrumental for breeding programs to select SBCMV-resistant genotypes in durum wheat, with the aim of increasing the frequency of *Sbm2*-resistant haplotype. This work paves the way for widening the investigation to bread wheat germplasm, where *Sbm2* has also been described.

Additionally, the effective development of *Sbm2* haplotype tagging KASP® presented herein can be considered as a case study to innovate plant variety testing in order to improve the efficacy and accuracy of European variety tests as well as the assessment of the value for cultivation and use and the decision-making process.

The recent availability of the genome assembly of the parental line and germplasm founder Svevo durum wheat allowed us to compare the genetic and physical maps at the target region and identify putative candidate genes. Therefore, this study provides the basis for further refining the *Sbm2* interval and paves the way for the positional cloning of this locus.

## Supplementary Information

Below is the link to the electronic supplementary material.

Supplementary file1 (DOCX 4017 kb)

Supplementary file2 (XLSX 37 kb)

Supplementary file3 (XLSX 155 kb)

Supplementary file4 (XLSX 95 kb)

Supplementary file5 (XLSX 21 kb)

Supplementary file6 (XLSX 26 kb)

## Data Availability

The datasets generated in the current study are available as Supplementary Information (SI). Additional datasets are available from the corresponding author on request.

## Abbreviations

BLUES: Best linear unbiased estimates
Cc: Cultivar Ciccio
Cl: Cultivar Claudio
Cv: Cultivar
DArT®: Diversity array technology marker
ELISA: Enzyme-linked immunosorbent assay.
GO: Gene ontology
GS: Genetic similarity
GWAS: Genome-wide association study
HC: Genes annotated at high confidence
IWGSC: International Wheat Genome Sequencing Consortium
IBS: Identity by state
IBD: Identity by descent
K: Kinship
KASP®: Kompetitive allele-specific PCR
KUBO: KASP® marker developed by DISTAL laboratory at University of Bologna
LC: Genes annotated at low confidence
MAS: Marker-assisted selection
MLM: Mixed linear model
Mr: Cultivar Meridiano
NJ: Neighbor joining tree
PVE: Proportion of variance explained
QTL: Quantitative trait locus
RIL: Recombinant inbred line mapping population
SBCMV: Soil-borne cereal mosaic virus
Sbm1: Soil-borne mosaic resistance locus 1.
Sbm2: Soil-borne mosaic resistance locus 2
SBWMV: Soil-borne wheat mosaic virus
SEV: Disease severity
SNP: Single-nucleotide polymorphism
Sv: Cultivar Svevo
