# Supplementary material for: Dissecting the genetic basis of resistance to Soil-borne cereal mosaic virus (SBCMV) in durum wheat by bi-parental mapping and GWAS
Source: Theor Appl Genet. 2024 Sep 2;137(9):213. doi: 10.1007/s00122-024-04709-7 (PMC11369050; doi:10.1007/s00122-024-04709-7)
Supplement: Supplementary file 1 — Supplementary file1 (DOCX 4017 kb) [file 122_2024_4709_MOESM1_ESM.docx]

**Dissecting the genetic basis of resistance to *Soil-Borne Cereal Mosaic Virus* in durum wheat by bi-parental mapping and GWAS.**

Martina Bruschi^1^, Matteo Bozzoli^1^, Claudio Ratti^1^, Giuseppe Sciara^1^, Ellen Goudemand^2^, Pierre Devaux^2^, Danara Ormanbekova^1^, Simona Corneti^1^, Sandra Stefanelli^1^, Sara Castelletti^1^, Elena Fusari^1^, Jad B. Novi^1^, Elisabetta Frascaroli^1^, Silvio Salvi^1^, Dragan Perovic^3^, Agata Gadaleta^4^, Concepcion Rubies-Autonell^1^, Maria Corinna Sanguineti^1^, Tuberosa Roberto^1^, Marco Maccaferri^1^

^1^Alma Mater Studiorum – Università di Bologna, Department of Agricultural and Food Sciences (DISTAL), University of Bologna, 40127 Bologna, Italy

^2^S.A.S. Florimond-Desprez Veuve and Fils, BP41, 59242, Cappelle-en-Pévèle, France

^3^Julius Kühn-Institut (JKI), Federal Research Centre for Cultivated Plants, Institute for Resistance Research and Stress Tolerance, Erwin-Baur-Str. 27, 06484, Quedlinburg, Germany

^4^Department of Soil, Plant and Food Science (Di.S.S.P.A.), University of Bari ‘Aldo Moro’, 70126 Bari, Italy

* For correspondence (email [marco.maccaferri@unibo.it](mailto:marco.maccaferri@unibo.it))

**ORCID iDs:**

Marco Maccaferri, <https://orcid.org/0000-0002-1935-3282>

***Author for correspondence**:

Marco Maccaferri, marco.maccaferri@unibo.it Tel.: +39-0512096645

**Supplemental Information (SI)**

**Supplemental Methods, Tables and Figures**

**Method S1. KASP® marker development protocol**

The developed KASP® markers were assayed on the parental lines of the RIL populations used in the study (*Mr*x*Cd* and *Sv*x*Cc*). Artificial heterozygous samples were produced by mixing the same amount of DNA of a resistant and of a susceptible parent. Each KASP® assay was tested on several replicates of the four parental lines and the heterozygous samples.

PCR for KASP® assays were set up in 96-wells optical plates in a total volume of 10 μL per reaction, containing 2 μL of genomic DNA at a concentration of 20 ng/μL, 5 μL of KASP master mix (2X), 0.14 μL of primer mix and 2.86 μL of Milli-Q water. Primer mix was composed by 12 μL of primer A, 12 μL of primer B, 30 μL of primer C (each primer at 100 μM) and 46 μL of Milli-Q water (total volume = 100 µL).

PCR reaction protocol and thermal cycling program were used as proposed by the “KASP genotyping chemistry User guide and manual” available at the website <https://www.lgcgroup.com/products/kasp-genotyping-chemistry> (PCR protocol was adjusted for the quantity of DNA/water per reaction). Primer mix was prepared as reported in Yuan et al. (2014).

The KASP® thermal cycling program used is reported herein**.**

To better discriminate allele separation, further recycling steps are needed repeating Step3 for additional 10 cycles.

Once the PCR thermal cycling program was completed, optical plates were read on an ABI 7500, Applied Biosystems^TM^ instrument. This stage of the process consisted in performing a thermal recycling program composed of three cycles as follows: denaturation at 95 °C for 30 sec, annealing/elongation at 55 °C for 60 sec and post-PCR read at 30 °C for 60 sec. The recycling program was repeated several times until three defined genotyping clusters were outlined in the output graphical plot.

Functional KASP® assays were manually selected based on the output graphical plot and on the correct clustering of controls.

Thermal cycling program used for KASP assays as proposed by the “KASP genotyping chemistry User guide and manual” available at the website <https://www.lgcgroup.com/products/kasp-genotyping-chemistry>

| **Stage** | **Temperature and length specifications** | **Cycle type and number** | **Number of cycles** |
| --- | --- | --- | --- |
| **1** | 94°C for 15 minutes | Hot-start activation | 1 |
| **2** | 94°C for 20 seconds | Denaturation | 10 |
|  | 61-55°C for 60 seconds | Annealing: dropping 0.6°C per cycle to reach a final annealing temperature of 55°C |  |
| **3** | 94°C for 20 seconds | Denaturation | 26 |
|  | 55°C for 60 seconds | Annealing |  |

**Method S2. Genotyping of Durum Panel and bi-parental cross RIL populations. Validation of KASP® markers**

Newly developed KASP® markers were used to genotype the Durum Panel UNIBO with the aim of validating the assays. DNA samples of the panel accessions were already available at the Plant Genetics Laboratory at UNIBO. PCR reactions were performed following the protocol previously described for KASP markers. The same accessions were genotyped with marker *wPt-2106*. PCR reactions for *wPt-2106* were set up in 96-well plates, in a total volume of 20 µL per reaction composed as following: 5 µL of genomic DNA at 20 ng/µL, 4 µL of buffer (5X), 1.6 µL of MgCl_2_, 0.16 µL of dNTPs, 0.05 µL of Taq Polymerase, 2.4 µL of primer mix and 6.79 µL of Milli-Q water. Primer mix was composed by 100 µL of primer forward Meridiano, 100 µL of primer forward Claudio and 100 µL of primer reverse (each primer at 100 µM) and 700 µL of Milli-Q water (total volume = 1 mL). The thermal cycling program used was the following: 5 min at 94 °C, 45 sec at 94° C, 45 sec at 60 °C, 1 min at 72 °C, 10 min at 72 °C and ∞ at 12 °C for 35 cycles. PCR products were electrophoresed in 2.5% agarose gel and visualized after staining with ethidium bromide. For each marker, two DNA samples of *Sv*, two of *Cc* and two of artificial *Sv-Cc*-heterozygous were used as control.

**Method S2 (continuation). Genotyping of Durum Panel and bi-parental cross RIL populations. Validation of KASP® markers**

**Genotyping of RIL population**

The *Sv* x *Cc* RIL population (3,075 lines) were genotyped with two distal KASP markers flanking the *QSbm.ubo-2BS* region, *KUBO-13* and *KUBO-9*, aimed at selecting RILs recombinant between the two markers. RILs DNA was isolated from lyophilized leaf tissue using a CTAB DNA isolation protocol for 96-well plates. PCR reactions were performed on the isolated RILs DNA following the protocol already described in previous section.

The *Sv* x *Cc* RILs selected through MAS were sown in two seasons (2015/2016 and 2015/2017) in a field experiment aimed at measuring the severity of SBCMV infection in Cadriano (Bologna, Italy, 44°35’ N 11°27’ E). The experimental design was a randomized complete block design with controls included in each block and was performed in two or three replicates depending on the season. Sowing density was 300 seeds/m^2^. Phenotyping and data analysis was carried out as described for the durum panel. Tables reporting scoring dates for Sv x Cc trial is showed in Supplementary material section (Supplemental Table 3).

RILs sown in the experimental fields were successively genotyped with seven markers located between the flanking markers *KUBO-3* and *KUBO-9* (i.e., *KUBO-1*, *KUBO-3*, *KUBO-27*, *KUBO-29*, *KUBO-40*, *KUBO*-*41* and *wPt-2106*) with the aim of selecting lines presenting a recombination inside the interested interval. An ELISA test was performed on the sole seven lines presenting a recombination event inside this interval, to measure the virus concentration in the leaves.

Based on the *Mr* x *Cl* linkage map from Maccaferri et al. (2015), 30 RILs presenting recombination between the SNPs used to design *KUBO-13* and *KUBO-9* or immediately outside this interval were selected and genotyped with KASP markers *KUBO-1*, *KUBO-3*, *KUBO-9*, *KUBO-13*, *KUBO-27*, *KUBO-29*, *KUBO-40* and *KUBO*-*41*.

Genotypic data obtained for *Sv* x *Cc* and *Mr* x *Cl* RIL populations were used to construct a genetic map for each population using the software JoinMap v. 4 (Van Ooijen, 2006). Marker order and genetic distances between markers were calculated using the Maximum Likelihood algorithm and the Haldane’s mapping function. The two maps were compared to check the order of KASP® markers in the region between *KUBO-13* and *KUBO-9*. Once markers order was determined, genotypic data were integrated with phenotypic data to evaluate the presence of informative recombination events between markers and to define the interval where *QSbm.ubo-2BS* was most probably confined. SBCMV resistance mapping by single-marker analysis was carried out in Windows QTL Cartographer v. 2.5 (Wang et al., 2012). Interval mapping (IM) analysis was also carried out to calculate the LOD score in the interval between *KUBO-13* and *KUBO-9*. Threshold value was set at 3.0 (rounded) via permutations. Walk speed was set at 0.5 cM.

**Validation of KASP® markers**

A comparison of the genotypic calls obtained with the Illumina 90K SNPs (data already available) and the newly developed KASP markers is reported in **Table S3**. Overall, the percentage of conflicts was low, with a maximum of 2.71% and 2.50 for *KUBO-40* and *KUBO-9* and zero conflicts for *KUBO-27*. The highest percentage of missing data resulted from *KUBO-1* (9.88%), while the lowest was detected for *KUBO-27* and *KUBO-29* (2.71%). Overall, *KUBO-27* seemed to be the most efficient among the developed markers. Wpt-2106 ASO and HRM assays performed as excellent markers.

**Method S3. Comparison of markers order in *Sbm2* region among genomes**

Marker order between *IWB73347* (*KUBO-13*, position: 9,986,862 bp) and IWB10512 (*KUBO-9* position: 24,313,794 bp) were compared in Svevo, in Chinese Spring bread wheat reference genome (CS, IWGSC RefSeq V2.1; Zhu et al., 2021) and in the wild emmer Zavitan genome (WEW_v2.0; Zhu et al., 2019). This physical region comprises *IWB10512* (*KUBO-9*, position on Svevo genome: 24,313,744), the marker flanking *Qsbm.ubo-2BS* along with *KUBO-13*.

Marker physical position on the durum wheat genome assembly (Maccaferri et al., 2019) was determined by blastn analysis. BLAST version 2.11.0 was run using the flanking marker sequences as queries with an e-value threshold of 10^-10^ threshold. To solve multiple hits issues due to homoelogs/paralogs matches, for each query, the best hit was defined as the hit with the smallest e-value and the longest High-scoring Segment Pair (HSP) alignment. Hits of markers in the confidence interval were manually cross-checked. Similar blast analyses were applied to determine the physical position of markers on the Wild Emmer Wheat Zavitan WEWSeq v2.0 (Zhu et al., 2019) and the Wheat Chinese Spring IWGSC RefSeq v2.1 (Zhu et al., 2021) genome assemblies.

The co-linearity among intervals was compared using MUMmer4 (“MUMmer4: A fast and versatile genome alignment system” Marçais et al., PLoS computational biology (2018), 14(1): e1005944.). Corresponding intervals for the three reference genomes were extracted using bedtools v2.30.0 software. The alignment was performed using *nucmer* command from *MUMmer4* software, using Svevo as reference genomes for the comparison with Chinese Spring and Zavitan.

Nucmer was used with the --mum parameter and the output *delta* file was filtered using the delta-filter command with the following options: -l 5000 to keep alignments longer that 5 kbp between reference and query and -r and -q, which retain only alignment that form the long consistent set for reference and query respectively. The filtered output was used to generate the plot with the *mummerplot* command and the following options: --large to set the output size, and --layout in order to orient and order the sequences such that the largest alignments hit the plot cluster near the main diagonal. The output plot shows the reference sequence (x axis) and the query sequence (y axis) where the bp distance is reported, if the alignment is colinear the dots are displayed in purple, whereas inversions are shown in blue.

**Method S4. Comparison of marker and gene order in** ***Sbm2* region among genomes**

A graphical comparison of marker order in *Sbm2* region among the three Zavitan, Svevo and Chinese Spring genomes is shown in **Figure S4**. Order inconsistencies among physical maps of the three genomes regarded 26 markers, mostly located in a few regions that showed rearrangements among assemblies, including a group of eight markers had their start position inverted between Svevo and Zavitan in a region of two kb in Svevo and of three kb in Zavitan (*IWB71947, IWB35524, IWB72156, IWB62546, IWB41644, IWB73884, IWB29097* and *IWB72157*)**.**

Two markers were positioned inconsistently among the three genomes (*IWB45152* and *wPt-1601*), one marker was positioned differently in Chinese Spring in respect to the other two genomes (*IWB28973*), three markers showed inconsistency in Zavitan in respect to the other two genomes (*IWB57923* and *IWA5772*) and up to twelve markers were positioned differently in Svevo in respect to the other two genomes (*wPt-2106, IWB50507, IWB71519, IWB45885, IWB4596, IWA7545, IWB26871, IWB26232, IWA3589, IWB26233, IWB65752, IWB8851*). These rearrangements occurred in a region of 6.5 – 8 Mb depending on the genome. A group of eight markers had their start position inverted between Svevo and Zavitan in a region of two kb in Svevo and of three kb in Zavitan (*IWB71947, IWB35524, IWB72156, IWB62546, IWB41644, IWB73884, IWB29097* and *IWB72157*).

The three wheat genome assemblies including Svevo, Chinese Spring (CS) v2.0, and Zavitan (Zv) v2.1 pseudomolecules were compared for collinearity in the interval *IWB73347* (*KUBO-13*, position: 9,986,862 bp) and *IWB10512* (KUBO 9, position: 24,313,794 bp), including *Sbm2* (**Figure S4**). As regards to Svevo (as reference) *versus* CS v2.0 (**Figure S5A**), assemblies are co-linear except for three main inversions on the Svevo in the intervals 10,631,152 -10,952,254 bp, 14,322,568-14,781,844 bp, and 17,971,448-18,480,002 bp. The first two inversions do not include molecular markers from Illumina 90K SNP Chip array. On the other hand, the third inversion includes the markers already detected with Pretzel software confirming those results, namely *IWB45885, IWB4596, IWA7545, IWB26871, IWB26232, IWA3589, IWB26233.* The random dots detected in the plot reported duplication or inversion of 5-7 kb size.

More differences among assemblies were detected using Zavitan as query and Chinese Spring or Svevo as references (**Figure S5B and S5C**). Both comparisons showed more duplications and alignment gaps. In addition, major inversions were detected between Zavitan and Svevo where two of them corresponded to the first and third inversions previously reported between Svevo and Chinese Spring. No inversions were detected between Zavitan and Chinese Spring assemblies, indicating that the local inversions in Svevo genomes could be artifacts.

**Table S1a.** Primers designed for KASP® assays. Each assay was composed of three primers: two allele-specific primers (A) and (B) and a common primer (C). Allele-specific primers A and B presented the probe sequence respectively for FAM (5’ GAAGGTGACCAAGTTCATGCT 3’) and HEX (5’ GAAGGTCGGAGTCAACGGATT 3’) at 5’ end.

| **SNP**  **ID** | **KASP® marker** | **Primer allele specific A (5’ FAM)** | **Primer allele specific B (5’ HEX)** | **Primer common C** |
| --- | --- | --- | --- | --- |
| *IWB28973* | *KUBO-1* | GAAGGTGACCAAGTTCATGCTcgtcttgttggtcagctgT | GAAGGTCGGAGTCAACGGATTcgtcttgttggtcagctgC | gcagctgtgacacgaacatta |
| *IWB28973* | *KUBO-38* | GAAGGTGACCAAGTTCATGCTccgtcttgttggtcagctgT | GAAGGTCGGAGTCAACGGATTccgtcttgttggtcagctgC | gcagagcagctgtgacacg |
| *IWB8328* | *KUBO-3* | GAAGGTGACCAAGTTCATGCTtgaagtggaggaactagaagcT | GAAGGTCGGAGTCAACGGATTtgaagtggaggaactagaagcC | gagaaggtgctgacatcattttc |
| *IWB24939* | *KUBO-5* | GAAGGTGACCAAGTTCATGCTcaaaatctggggcatgctcA | GAAGGTCGGAGTCAACGGATTcaaaatctggggcatgctcG | agagctagggtggtttcagg |
| *IWB6204* | *KUBO-6* | GAAGGTGACCAAGTTCATGCTgacaaagttggtggtaaattctcttaC | GAAGGTCGGAGTCAACGGATTggacaaagttggtggtaaattctcttaT | gcaagcatgccttccaccaccaa |
| *IWB10512* | *KUBO-8* | GAAGGTGACCAAGTTCATGCTatcagaatatgtacaattattatgccaagaT | GAAGGTCGGAGTCAACGGATTcagaatatgtacaattattatgccaagaC | ggcggaaattctcttcctttccttattta |
| *IWB10512* | *KUBO-9* | GAAGGTGACCAAGTTCATGCTaggatgcgttgtttgccaactctA | GAAGGTCGGAGTCAACGGATTggatgcgttgtttgccaactctG | gccatccaccaccaccataagtta |
| *IWB73347* | *KUBO-12* | GAAGGTGACCAAGTTCATGCTggctgacagggacattgcT | GAAGGTCGGAGTCAACGGATTggctgacagggacattgcC | ctgtaaacggactctcgaca |
| *IWB73347* | *KUBO-13* | as KUBO-012_A | as KUBO-012_B | gtgcatttcaaaattcattttctc |
| *IWB73347* | *KUBO-14* | GAAGGTGACCAAGTTCATGCTgcatctctgacattctcagaacA | GAAGGTCGGAGTCAACGGATTgcatctctgacattctcagaacG | cggggacggtttcagg |
| *IWB61884* | *KUBO-15* | GAAGGTGACCAAGTTCATGCTaaggagtgaaaccaagcgtaT | GAAGGTCGGAGTCAACGGATTaaggagtgaaaccaagcgtaC | caccaactctgacactggc |
| *IWB61884* | *KUBO-16* | as KUBO-015_A | as KUBO-015_B | tgacatcacccaccttatcgt |
| *IWB61884* | *KUBO-17* | GAAGGTGACCAAGTTCATGCTccccaagcgctgacG | GAAGGTCGGAGTCAACGGATTccccaagcgctgacA | ggagcatggtatgacagacatc |
| *IWB11421* | *KUBO-26* | GAAGGTGACCAAGTTCATGCTtaaacaatcactcgctaataagcaaC | GAAGGTCGGAGTCAACGGATTtaaacaatcactcgctaataagcaaT | aaagtaggtgcacagcctgag |
| *IWB11421* | *KUBO-27* | as KUBO-026_A | as KUB-O26_B | gccatatgctgtgactgctg |
| *IWB23029* | *KUBO-29* | GAAGGTGACCAAGTTCATGCTgctggaggaactagaagctgaG | GAAGGTCGGAGTCAACGGATTgctggaggaactagaagctgaT | aacagagcattgcaaaaccta |
| *IWB23029* | *KUBO-31* | as KUB-O29_A | as KUB-O29_B | ggagtcagttggtgcattcg |
| *IWB29097* | *KUBO-40* | GAAGGTGACCAAGTTCATGCTGaTGCTcctTtTaTcccaggaA | GAAGGTCGGAGTCAACGGATTGaTGCTcctTtTaTcccaggaG | tcgaatcatgaggacctggg |
| *IWB35524* | *KUBO-41* | GAAGGTGACCAAGTTCATGCTtgtGcattgtgaaggtgatttaaA | GAAGGTCGGAGTCAACGGATTtgtGcattgtgaaggtgatttaaG | acttgcagacgaaccatgtag |
| *IWB23330* | *KUBO-4* | GAAGGTGACCAAGTTCATGCTtgcaccccttgttctttgaaT | GAAGGTCGGAGTCAACGGATTtgcaccccttgttctttgaaC | aaacaagacaagataatgcaagtct |
| *IWB10512* | *KUBO-7* | GAAGGTGACCAAGTTCATGCTaggatgcgttgtttgccaactctA | GAAGGTCGGAGTCAACGGATTggatgcgttgtttgccaactctG | gccatccaccaccaccataagtta |
| *IWB8390* | *KUBO-10* | GAAGGTGACCAAGTTCATGCTcctctgcaacgccgccgC | GAAGGTCGGAGTCAACGGATTcctctgcaacgccgccgT | tatgcgggtcggcgatgacgtt |
| *IWB8390* | *KUBO-11* | GAAGGTGACCAAGTTCATGCTagagtcaaggaactcccacG | GAAGGTCGGAGTCAACGGATTagagtcaaggaactcccacA | gtgtgaatgagagaatctttgacg |
| *IWB8390* | *KUBO-12* | GAAGGTGACCAAGTTCATGCTccgatactaatcctaggaattacgG | GAAGGTCGGAGTCAACGGATTccgatactaatcctaggaattacgA | aggaagtcatgagtccttggta |
| *IWB11421* | *KUBO-28* | as KUBO-026_A | as KUBO-026_B | tttctgcacctgccatatgc |
| *IWB23029* | *KUBO-30* | as KUBO-029_A | as KUBO-029_B | cgtcgagaagaatttgacagaa |
| *IWB42660* | *KUBO-32* | GAAGGTGACCAAGTTCATGCTttggcctgtaaaaggctatcG | 5' HEX GAAGGTGACCAAGTTCATGCTttggcctgtaaaaggctatcA | gatgagcataatctacttcctgatg |
| *IWB42660* | *KUBO-33* | as KUBO-032_A | as KUBO-032_B | tcctgatgccctagagagtgac |
| *IWB42660* | *KUBO-34* | as KUBO-032_A | as KUBO-032_B | ctatgttctcaatagtcatgaggc |
| *IWB41644* | *KUBO-35* | GAAGGTGACCAAGTTCATGCTaagtgcaggcatgtgttgagataA | GAAGGTCGGAGTCAACGGATTaagtgcaggcatgtgttgagataC | cggcgacattactgtcttaacc |
| *IWB41644* | *KUBO-36* | as KUBO-035_A | as KUBO-035_B | ggctggagcttgactgagaat |
| *IWB41644* | *KUBO-37* | as KUBO-035_A | as KUBO-035_B | ccagccctgctcctgatc |
| *IWB28973* | *KUBO-39* | GAAGGTGACCAAGTTCATGCTgaggcgtcgtgctgcctA | GAAGGTCGGAGTCAACGGATTgaggcgtcgtgctgcctG | tcgcaccgcatgtatggt |
| *IWB45152* | *KUBO-2* | GAAGGTGACCAAGTTCATGCTacaaacgaagaacaacacggT | GAAGGTCGGAGTCAACGGATTacaaacgaagaacaacacggC | tcgccctcccctttcctg |
| *IWB45152* | *KUBO-42* | as KUBO-002_A | as KUBO-002_B | ccctttcctggtgacagtga |

^a^SNP ID = ID code of the SNP on which the KASP® assay was developed.

^b^KASP® marker = name and number of the developed KASP® assay developed at UNIBO.

**Table S1b.** Primers designed for the DART marker wPt-2106 assay converted to allele-specific oligonucleotide PCR and high-resolution melting PCR.

| Primer name | Oligonucleotide sequence |
| --- | --- |
| *wPt-2106* Allele Specific Oligonucleotide (ASO, separation in horizontal electrophoresis) | |
| wPt-2106_ASO_Forward_Resistant | GCAACATCCTCTTGCAGGCA |
| wPt-2106_ASO_Forward_susceptible (M13 tail) | CACGACGTTGTAAAACGACATCCTCTTGCAGGCGGAA |
| wPt-2106_Reverse_common | CGCATAGAACATTATAAAGGCTGGC |
| *wPt-2106* high resolution melting (HRM) |  |
| wPt-2106_HRM_Forward | GGAGACCAGAGGAGGAACTTT |
| wPt-2106_HRM_Reverse | AAAACGGCGGTTCAGAAG |

**Table S2** Accuracy of KUBO KASP® markers versus the original Illumina 90K SNP on the UNIBO Durum Panel (% calculated on 258 DNA samples).

| **Marker** | **KUBO-13** | **KUBO-27** | **KUBO-29** | **KUBO-1** | **KUBO-3** | **KUBO-41** | **KUBO-40** | **KUBO-9** | **wPt-2106_**  **ASO** | **wPt-**  **2106_**  **HRM** |
| --- | --- | --- | --- | --- | --- | --- | --- | --- | --- | --- |
| Conflicts (%) | 1.55 | 0.00 | 0.78 | 0.39 | 1.16 | 1.55 | 2.71 | 2.50 | 0.50 | 0.65 |
| Missing (%) | 5.04 | 2.71 | 2.71 | 9.88 | 9.30 | 3.10 | 8.72 | 4.07 | 1.60 | 1.52 |

**Table S3:** Scoring dates for SBCMV symptom severity evaluation of the RIL population Sv x Cc in years 2016 and 2017 in Cadriano (Bologna, Italy). For each field, dates in which the evaluation was performed are marked with an “X”.

|  |  |  |  |  |  |
| --- | --- | --- | --- | --- | --- |
| Sv x Cc trials | Visual score dates | | | | |
|  | 2016 |  |  |  |  |
|  | March 14^th^ | March 17^th^ | March 30^th^ |  |  |
| Field 1 (Cadriano, Sv × Cc RIL) | x |  | x |  |  |
| Field 2 (Cadriano, Sv × Cc RIL) |  | x | x |  |  |
|  | 2017 |  |  |  |  |
|  | March 17^th^ | March 21^st^ | March 23^rd^ | March 28^th^ | April 3^rd^ |
| Field 1 (Cadriano, Sv × Cc RIL) | x | x |  | x | x |
| Field 2 (Cadriano, Sv × Cc RIL) |  |  | x | x | x |

**Table S4.** Peak markers of minor QTLs other than Sbm2 resulting from GWAS performed on Durum Panel UNIBO with BLUEs of spatially corrected visual score (SEV) (A) and ELISA (B) data collected in Cadriano (Bologna, Italy) in 2005, 2007 and 2010. QTL peaks confirmed by the SEV covariate analysis (Table 11) are in italic. Common QTL peaks between SEV and ELISA are in bold. Genetic positions (cM) are reported as in the consensus map of Maccaferri et al. (2015). Physical positions (bp) are reported as the start positions obtained by blasting the marker sequence on the durum wheat genome reference Svevo (Maccaferri et al., 2019). The Strand defines the direction of the marker sequence.

**Table S4a. Minor QTL for disease severity (SEV) score**

| Marker | Chr | Position (cM) | Position (bp) | Strand | -Log10 (P) | R^2^ (%) | Allele | Allele frequency | Associated significant markers | Interval (cM) |
| --- | --- | --- | --- | --- | --- | --- | --- | --- | --- | --- |
| IWB6984 | 1A | 98.2 | 526,410,713 | + | 3.45 | 4.1 | C/T | 0.75/0.25 | IWB26708, IWB11161, IWB11161, IWB12454, IWB60140, IWB6995, IWB31771 | 98.2-99.8 |
| **IWB71381** | **2A** | **206.5** | **768,804,948** | **-** | **3.56** | **4.3** | **A/G** | **0.66/0.34** | **IWB6580, IWB71583, IWB71381, IWB1939** | **206.2-206.8** |
| IWB6584 | 2B | 12.3 |  |  |  |  |  |  |  |  |
| **IWB48240** | **2B** | **67.6** | **109,386,560** | **+** | **3.78** | **4.6** | **C/T** | **0.29/0.71** | **IWB45048, IWB59095, IWB24241** | **67.6-68.7** |
| IWB27191 | 3B | 163.2 |  |  |  |  |  |  |  |  |
| IWA603 | 4A | 35.1 | 37,138,471 | - | 3.36 | 4.0 | A/G | 0.86/0.14 | IWA603, IWB71863, | 35.1-36.1 |
| **IWB6276** | **4A** | **147.4** | **698,244,266** | **-** | **4.37** | **5.5** | **A/G** | **0.43/0.57** | **IWB7898, IWA7765,** **IWB22582, IWB73460,** **wPt-4596, IWB47812,** **IWB8487,** **IWB60429,** **IWB73853,** **IWB11713,** **IWB5747,** **IWB9122** | **147.0-154.0** |
| **IWB37657** | **4A** | **156.90** | **707,180,653** | **-** |  |  | **A/G** | **0.52/0.48** | **IWB37657,** **wPt-9162, wPt-2331,** **IWB42403,** **IWB59017, IWB7370,** **IWB24067,** **IWB5461,** **IWB61457** | **156.9-160.2** |
| IWB14680 | 5A | 194.9 | 639,652,609 | + | 3.35 | 4.0 | A/G | 0.42/0.58 | IWB7282, IWB71385, IWB4836, IWB14445, IWB4261 | 194.9-199.0 |
| ***IWB67284*** | ***5B*** | ***53.8*** | ***411,136,220*** | ***+*** | ***3.77*** | ***4.6*** | ***C/T*** | ***0.63/0.37*** | ***IWA6526, IWB69519, IWB72308, IWB70171,*** ***IWB71912,*** ***IWB33231,*** ***IWA5742, IWB35030*** | ***52.2-59.2*** |
| **WPt-9589** | **6B** | **153.8** | **689678232** | **/** |  |  | **+/-** | **0.69/31** | **IWA1046, wPt-6116** | **153.1-153.8** |
| IWB67175 | 7A | 14.1 |  |  | 4.37 | 5.5 | A/G | 0.64/0.36 | IWB13845, IWB26796, IWB4929, IWB54678, IWB68596, IWB68597, IWB71783, IWB72082, IWB67174 | 14.1-14.2 |
| IWB64582 | 7A | 30.9 | 30,563,270 | + | 4.44 | 5.6 | A/G | 0.08/0.92 | IWB44377, IWB65343 | 30.9-30.9 |
| **IWB72581** | **7A** | **102.4** | **147,862,030** | **-** | **3.35** | **4.0** | **C/T** | **0.14/0.86** | **IWA7500,** **IWB11693,** **IWB34318,** **IWB34718,** **IWB34968, IWB43994,** **IWB60270,** **IWB60652,** **IWB30521, IWB67852,** **IWB71715,** **IWB72581** | **100.6-102.4** |
| **IWB71789** | **7B** | **146.2** | **703,441,815** |  |  |  | **T/C** | **0.60/040** | **IWB68926,** **IWB73350,** **IWB72357,** **wPt-3533, IWB70061, IWB41479, IWB41483, IWB71789, IWB9853** | **142.5-147.0** |
| wPt-7191 | 7B | 159.7 | 669,481,736 | / | 3.55 | 4.3 | A/T | 0.60/0.40 | wPt-7351, IWB68458, IWA2369, IWA6532, IWB7099 | 157.0-161.7 |

**Table S4b. Minor QTL for disease ELISA value**

| Marker | Chr | Position (cM) | Position (bp) | Strand | -Log10(P) | R^2^ (%) | Allele | Allele frequency | Associated significant markers | Interval (cM) |
| --- | --- | --- | --- | --- | --- | --- | --- | --- | --- | --- |
| **IWB71381** | **2A** | **206.5** | **768,804,948** | **-** | **3.56** | **4.3** | **A/G** | **0.66/0.34** | **IWB6580, IWB71583, IWB71381, IWB1939** | **206.2-206.8** |
| IWB6584 | 2B | 12.3 |  |  |  |  |  |  |  |  |
| **IWB48240** | **2B** | **67.6** | **109,386,560** | **+** | **3.78** | **4.6** | **C/T** | **0.29/0.71** | **IWB45048, IWB59095, IWB24241** | **67.6-68.7** |
| **IWB41739** | **2B** | **127.6** | **126,353,132** |  |  |  |  |  | **IWB10507, IWB38099, IWB39865, IWB6524, IWB73199, IWB47474, IWB71648, IWB2008, IWB41739, IWB72409, IWB72410** | **126.6-127.6** |
| IWB26062 | 4A | 41.3 | 47,970,400 |  |  |  |  |  | IWB43503, IWB48136, IWB48137, IWB50371, IWB65359, IWB26062, IWA7521, IWA7537, IWB26513 | 41.3-42.7 |
| **IWB6276** | **4A** | **147.4** | **698,244,266** | **-** | **4.37** | **5.5** | **A/G** | **0.43/0.57** | **IWB7898, IWA7765,** **IWB22582, IWB73460,** **wPt-4596,** **IWB47812, IWB8487,** **IWB60429,** **IWB73853,** **IWB11713,** **IWB5747,** **IWB9122** | **147.0-154.0** |
| **IWB42403** | **4A** | **156.90** |  |  |  |  | **A/G** | **0.52/0.48** | **IWB37657,** **wPt-9162,** **wPt-2331,** **IWB42403, IWB59017,** **IWB7370,** **IWB24067,** **IWB5461, IWB61457** | **156.9-160.2** |
| IWB71493 | 4B | 59.5 | 481,368,463 | + | 3.77 | 5.3 | G/T | 0.18/0.82 | IWB29213, IWB31142, IWB70363, IWB72279, IWB28921, IWB67265, IWB73838 | 56.1-60.4 |
| ***IWB8425*** | ***6B*** | ***145.3*** | ***681,667,256*** | ***+*** | ***3.42*** | ***4.7*** | ***C/T*** | ***0.19/0.81*** | ***IWB13510, IWB42949, IWB5463, IWB57608, IWB74861, IWB8425, wPt-1325, IWB26763, IWB9416*** | ***145.3-145.9*** |
| **WPt-9589** | **6B** | **153.8** |  |  |  |  | **+/-** | **0.69/31** | **IWA1046,** **wPt-6116,** | **153.1-153.8** |
| IWB63084 | 7A | 90.2 | 113,472,467 | + | 4.68 | 6.8 | A/G | 0.47/0.53 | IWB4104, IWB47061, IWB53919, IWB65392, IWB65659, IWB72815, IWB72952, IWB62086, IWB63084, IWB26977, IWB62888, IWB72813 | 89.6-92.9 |
| **IWB72581** | **7A** | **102.4** | **147,862,030** | **-** | **3.35** | **4.0** | **C/T** | **0.14/0.86** | **IWA7500, IWB11693, IWB34318,** **IWB34718,** **IWB34968, IWB43994, IWB60270, IWB60652, IWB30521,** **IWB67852,** **IWB71715,** **IWB72581** | **100.6-102.4** |
| **IWB71789** | **7B** | **146.2** |  |  |  |  | **T/C** | **0.60/040** | **IWB68926,** **IWB73350,** **IWB72357,** **wPt-3533, IWB70061,** **IWB41479,** **IWB41483,** **IWB71789,** **IWB9853** | **142.5-147.0** |

**Table S5.** Population structure effect and GLM full model including *Sbm2* and minor QTL for SBCMV response including symptom severity (SEV) and virus quantification in leaves (ELISA). Analysis based on the UNIBO Durum Panel of 258 worldwide cultivars and breeding lines. *P* values and *R*^2^ are presented for the structure alone and for *Sbm2* and the additional 21 putative minor QTL, and their interactions with *Sbm2*.

|  |  | **SEV** |  |  |  | **ELISA** |  |  |
| --- | --- | --- | --- | --- | --- | --- | --- | --- |
|  | **cM** | **p-value** |  | ***R*^2^** (%) |  | **p-value** |  | ***R*^2^ (%)** |
| **Q1_Italian_ICARDA_old (WestAsian_Haurani_Founder)** |  | 7.35E-01 | NS | 0.04 |  | 2.7E-01 | NS | 0.48 |
| **Q2_ICARDADryland**  **(Omrabi group)** |  | 8.45E-02 | NS | 1.16 |  | 4.3E-02 | * | 1.59 |
| **Q3_ICARDA_temperate** |  | 3.42E-05 | **** | 6.50 |  | 5.3E-05 | **** | 6.19 |
| **Q4_CIMMYT60_ICARDA**  **(YoriC69_Founder)** |  | 2.37E-01 | NS | 0.55 |  | 4.5E-01 | NS | 0.22 |
| **Q5_CIMMYT70**  **(YavarosC79_Founder)** |  | 1.03E-03 | ** | 4.13 |  | 1.0E-02 | * | 2.55 |
| **Q6_Italian**  **(Valnova_Founder)** |  | 1.14E-01 | NS | 0.97 |  | 2.2E-01 | NS | 0.58 |
| **Q7_CIMMYT80**  **(AltarC84_Founder)** |  | 9.36E-06 | **** | 7.40 |  | 1.8E-02 | * | 2.15 |
| **Q8_NorthAmerica_France** |  | 1.09E-03 | ** | 4.09 |  | 1.7E-02 | * | 2.19 |
| **Population structure Global *R*^2^** |  |  |  | **21.50** |  |  |  | **13.26** |
| IWB6984_1A | 98.2 | 3.48E-05 | **** | 6.49 |  | 2.17E-03 | ** | 3.61 |
| IWB71381_2A | 206.5 | 2.35E-02 | * | 1.99 |  | 1.69E-01 | NS | 0.74 |
| IWB6584_2B (=*Sbm2*) | 12.3 | 8.08E-46 | **** | 54.62 |  | 4.47E-37 | **** | 46.9 |
| IWB48240_2B | 67.6 | 3.99E-01 | NS | 0.28 |  | 1.87E-01 | NS | 0.68 |
| IWB41739_2B | 127.6 | 4.59E-01 | NS | 0.21 |  | 3.23E-02 | * | 1.78 |
| IWB27191_3B | 163.2 | 2.32E-02 | * | 2.00 |  | 5.87E-02 | * | 1.39 |
| IWA603_4A | 35.1 | 1.33E-06 | **** | 8.74 |  | 5.34E-02 | * | 1.45 |
| IWB26062_4A | 41.3 | 2.70E-01 | NS | 0.47 |  | 1.73E-01 | NS | 0.72 |
| IWB6276_4A | 147.4 | 1.50E-04 | *** | 5.47 |  | 7.66E-04 | *** | 4.33 |
| IWB37657_4A | 156.9 | 8.62E-03 | ** | 2.66 |  | 1.22E-02 | * | 2.43 |
| IWB42403_4A |  | 5.15E-03 | ** | 3.02 |  | 3.08E-04 | *** | 4.97 |
| IWB71493_4B | 59.5 | 2.84E-02 | * | 1.86 |  | 2.72E-04 | *** | 5.05 |
| IWB14680_5A | 194.9 | 7.70E-05 | **** | 5.93 |  | 9.38E-03 | ** | 2.61 |
| IWB67284_5B | 53.8 | 4.94E-04 | *** | 4.64 |  | 4.50E-03 | ** | 3.11 |
| IWA6276_6A | - | 4.39E-03 | ** | 3.13 |  | 6.56E-02 | * | 1.32 |
| IWB8425_6B | 145.3 | 4.75E-03 | ** | 3.07 |  | 1.78E-04 | *** | 5.35 |
| wPt-9589_6B | 153.8 | 1.01E-01 | NS | 1.05 |  | 5.36E-02 | * | 1.45 |
| IWB67175_7A | 14.1 | 9.69E-01 | NS | 0.01 |  | 9.24E-01 | NS | 0.01 |
| IWB64582_7A | 30.9 | 8.67E-03 | ** | 2.66 |  | 8.26E-02 | * | 1.17 |
| IWB63084_7A | 90.2 | 4.27E-01 | NS | 0.25 |  | 8.75E-03 | ** | 2.65 |
| IWB72581_7A | 102.4 | 6.28E-03 | ** | 2.88 |  | 1.72E-02 | * | 2.20 |
| IWB71789_7B | 146.2 | 2.99E-03 | ** | 3.39 |  | 9.70E-04 | *** | 4.17 |
| IWB6584* IWB6984_1A |  | 1.28E-01 | NS | 0.68 | * IWB6984_1A | 1.75E-02 | * | 1.17 |
| IWB6584* IWB71381_2A |  | 7.63E-01 | NS | 1.19 | * IWB41739_2B | 1.31E-01 | NS | 1.79 |
| IWB6584* IWB27191_3B |  | 1.33E-01 | NS | 0.65 | * IWB27191_3B | - |  |  |
| IWB6584_2B*IWA603_4A |  | 1.47E-02 | * | 2.90 | * IWA603_4A | - |  |  |
| IWB6584_2B*IWB6276_4A |  | 7.23E-03 | ** | 1.92 | * IWB6276_4A | 8.58E-01 | NS | 0.46 |
| IWB6584_2B*IWB37657_4A |  | 1.87E-06 | **** | 4.41 | * IWB37657_4A | 1.37E-01 | NS | 1.01 |
| IWB6584_2B*IWB42403_4A |  | 6.58E-02 | * | 1.20 | * IWB42403_4A | 2.17E-01 | NS | 2.12 |
| IWB6584_2B*IWB71493_4B |  | 5.15E-01 | NS | 0.18 | * IWB71493_4B | 5.83E-01 | NS | 1.74 |
| IWB6584_2B*IWB14680_5A |  | 3.47E-02 | * | 2.38 | * IWB14680_5A | 8.90E-01 | NS | 0.28 |
| IWB6584_2B*IWB67284_5B |  | 1.09E-01 | NS | 1.32 | * IWB67284_5B | 9.17E-05 | **** | 3.48 |
| IWB6584_2B*IWA6276_6A |  | 1.36E-02 | * | 1.20 | * IWA6276_6A | - |  |  |
| IWB6584_2B*IWB8425_6B |  | 7.00E-01 | NS | 0.06 | * IWB8425_6B | 8.02E-01 | NS | 0.79 |
| IWB6584_2B*IWB64582_7A |  | 3.75E-01 | NS | 1.32 | *IWB63084_7A | 1.01E-02 | * | 1.82 |
| IWB6584_2B*IWB72581_7A |  | 1.27E-01 | NS | 0.67 | *IWB72581_7A | 1.45E-01 | NS | 0.59 |
| IWB6584_2B* IWB71789_7B |  | 4.55E-01 | NS | 0.60 | * IWB71789_7B | 6.74E-01 | NS | 1.03 |
| **Global R^2^ (single markers, no pop structure as covariate)** |  |  |  | **62.3** |  |  |  | **52.1** |
| **Global R^2^ (single markers and marker interactions, no pop structure as covariate)** |  |  |  | **67.5** |  |  |  | **54.7** |

**Table S6.** Genotyping results of Sv × Cc and Mr × Cl RILs carrying recombinations in *Sbm2* region.

**Table S6a.** Genotyping results of Sv × Cc RILs carrying recombinations in Sbm2 region. A) Genotyping results of Sv × Cc RILs selected by MAS; B) Genotyping and phenotypic results of RILs carrying a recombination event between KUBO-27 and KUBO-40. A) and B) Phenotypic values are represented in a color scale from red (susceptible) to green (resistant). The red square indicates the interval where most probably Sbm2 is located.

^a^ Marker name,

^b^ Genetic positions (cM) reported as calculated by the software JoinMap,

^c^ Physical positions (bp) obtained by blasting the marker sequence on the durum wheat genome reference Svevo (Maccaferri et al., 2019),

^d^ Direction of the maker sequence,

^e^ SEV BLUEs data collected in 2016 (3 dates) and 2017 (5 dates) in Cadriano (Bologna, Italy) for Sv × Cc RILs and spatially corrected according to the moving mean of genotypic effects model,

^f^ B = Cc allele = susceptible,

^g^ A = Sv allele = resistant.

^h^ Number of individuals presenting a specific haplotype.

^i^ ELISA data obtained from sample of informative Sv × Cc RILs (2 replicates) collected in Cadriano (Bologna, Italy) in 2016 and 2017.

**Table S6b.** Genotyping results of Mr × Cl RILs carrying recombinations in Sbm2 region. C) Genotyping results of Mr × Cl RILs selected by MAS. Phenotypic values are represented in a color scale from red (susceptible) to green (resistant). Red square indicates the interval where most probably Sbm2 is located.

^a^ Marker name,

^b^ Genetic positions (cM) reported as calculated by the software JoinMap,

^c^ Physical positions (bp) obtained by blasting the marker sequence on the durum wheat genome reference Svevo (Maccaferri et al., 2019),

^d^ Direction of the maker sequence,

^e^ BLUEs of Symptom Severity scores based on phenotypic data of Maccaferri et al. (2011) adjusted according to the method used to record Sv × Cc symptom severity scores (for ease of comparison),

^f^ Number of individuals presenting that specific haplotype,

^g^ A = *Mr* allele = resistant,

^h^ B = Cl allele = susceptible.

**Table S7.** Description and number of genes associated to each description present in the support interval of Sbm2, based on the physical map of the Svevo genome.

******


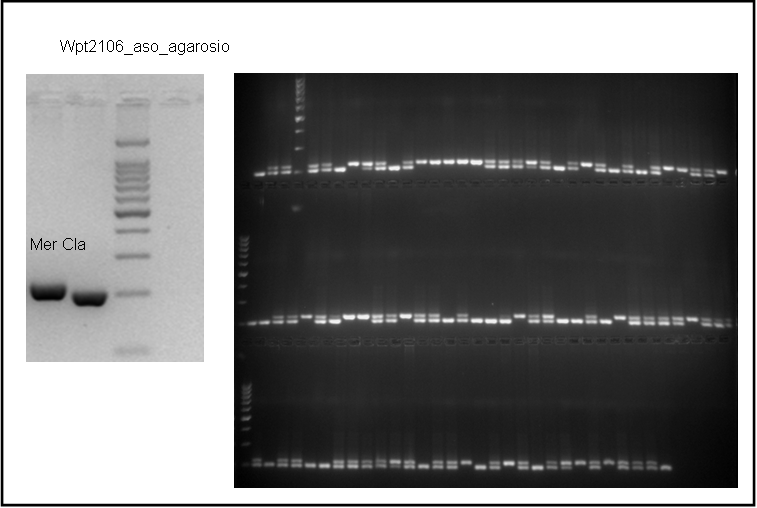


**Figure S1a.** Agarose and high-resolution melting PCR assays obtained for the DArT^®^ *wPt2106,* tagging *Sbm2* in durum wheat.

***wPt-2106_ASO*,** Allele Specific Oligonucleotide PCR assay for separation in standard horizontal electrophoretic gels.


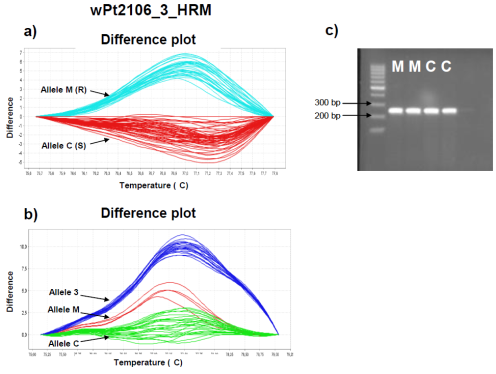


**Figure S1b.** Agarose and high-resolution melting PCR assays obtained for the DArT^®^ *wPt2106,* tagging *Sbm2* in durum wheat.

***wPt2106_HRM***, high resolution melting assay, for fluorescent detection.


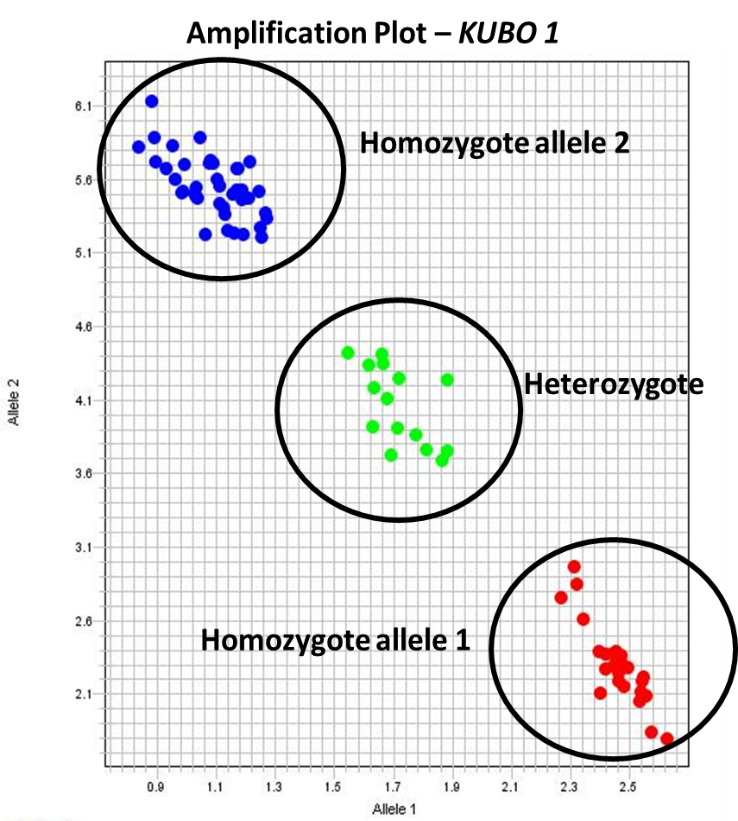

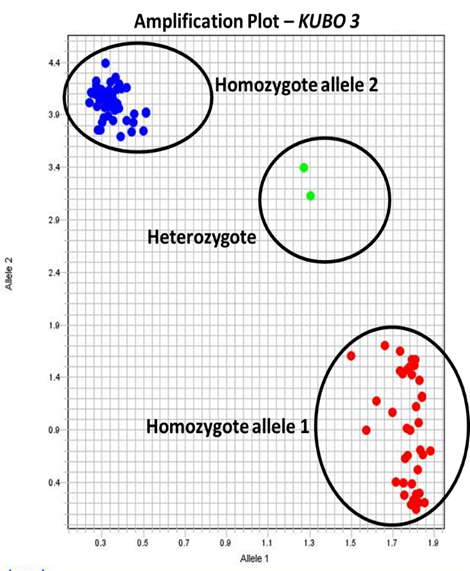

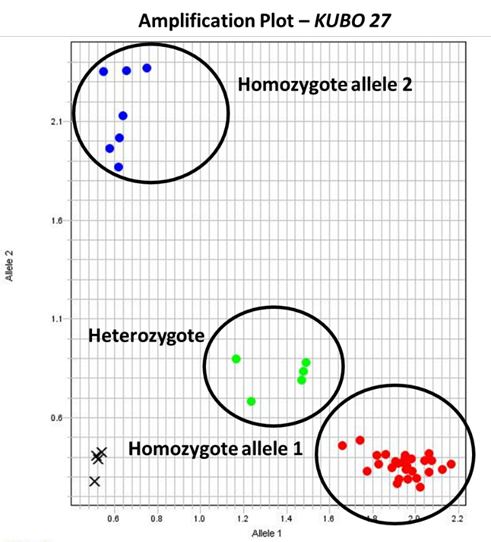

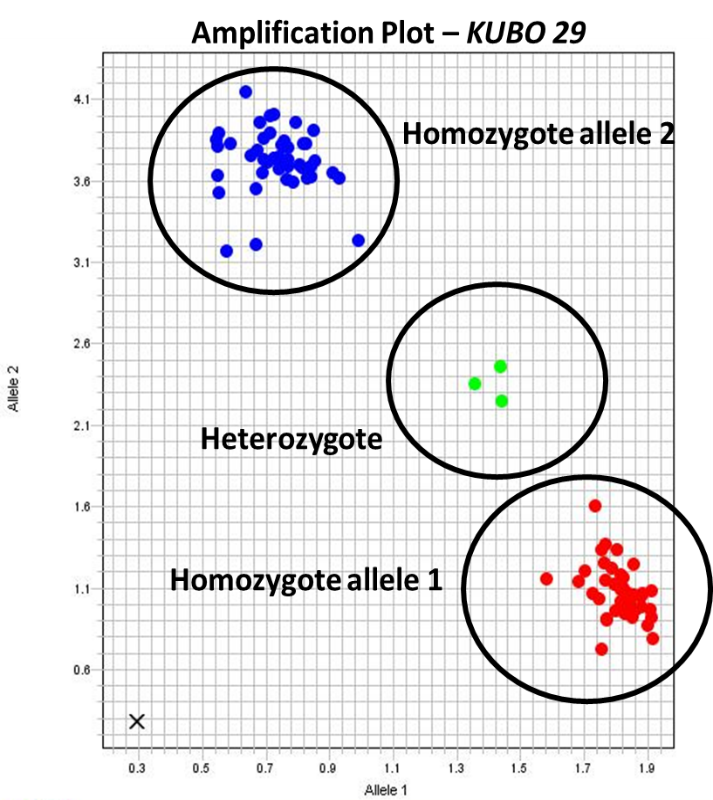


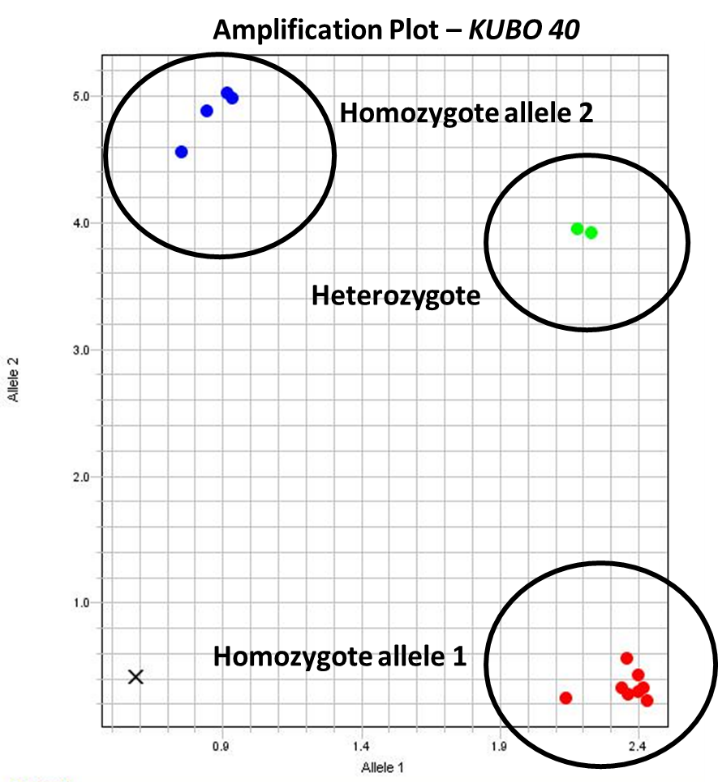

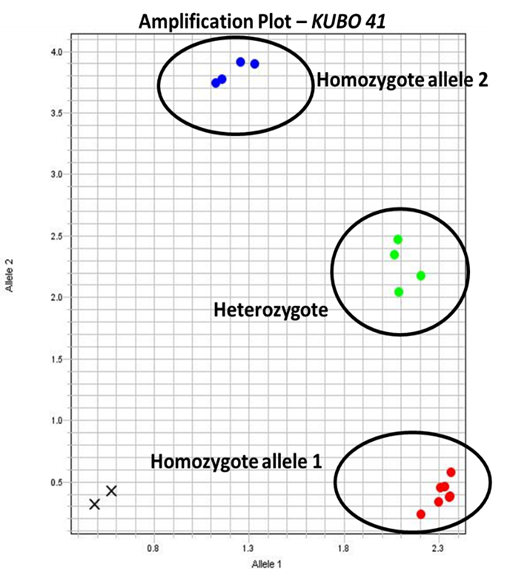


**Figure S2.** Amplification plots for *KUBO-1*, *KUBO-3*, *KUBO27, KUBO-29, KUBO-40*, *KUBO-41* KASP^®^ assays developed by UNIBO laboratory and obtained from the corresponding haplotype tagger Illumina SNP IWB28973, IWB8328, IWB11421, IWB23029, IWB29097 and IWB35524, respectively.


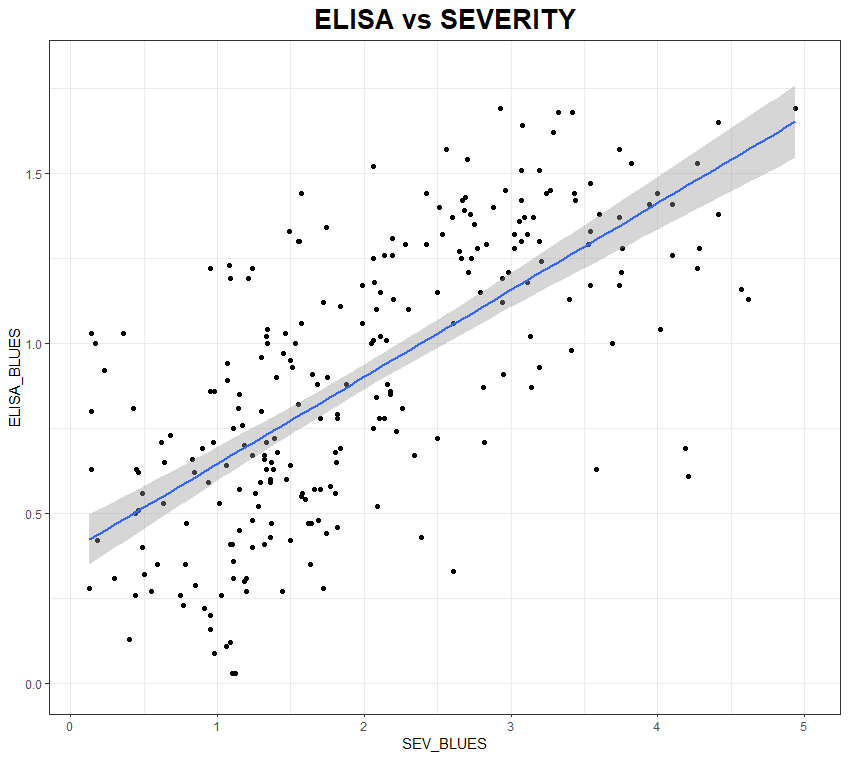


***r* = 0.69**

**Figure S3:** scatterplot of ELISA *versus* Visual severity (SEV) BLUES data across years obtained from the UNIBO Durum Panel of 258 cultivars and breeding lines, as evaluated in Cadriano SBCMV nursery.


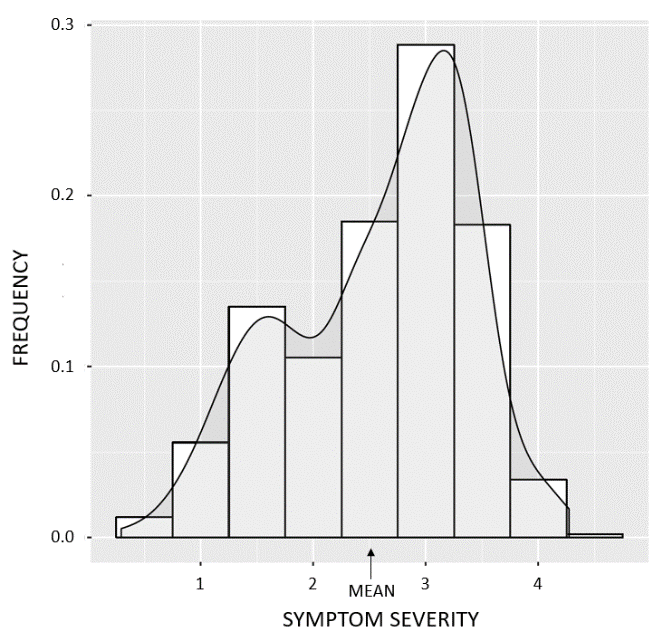


**Figure S4.** Frequency distribution of BLUEs obtained from symptom severity scores (SEV) data collected in 2016 (3 dates) and 2017 (5 dates) in Cadriano (Bologna, Italy) for Sv × Cc RILs. Field data were spatially corrected according to the moving mean of genotypic effects model.


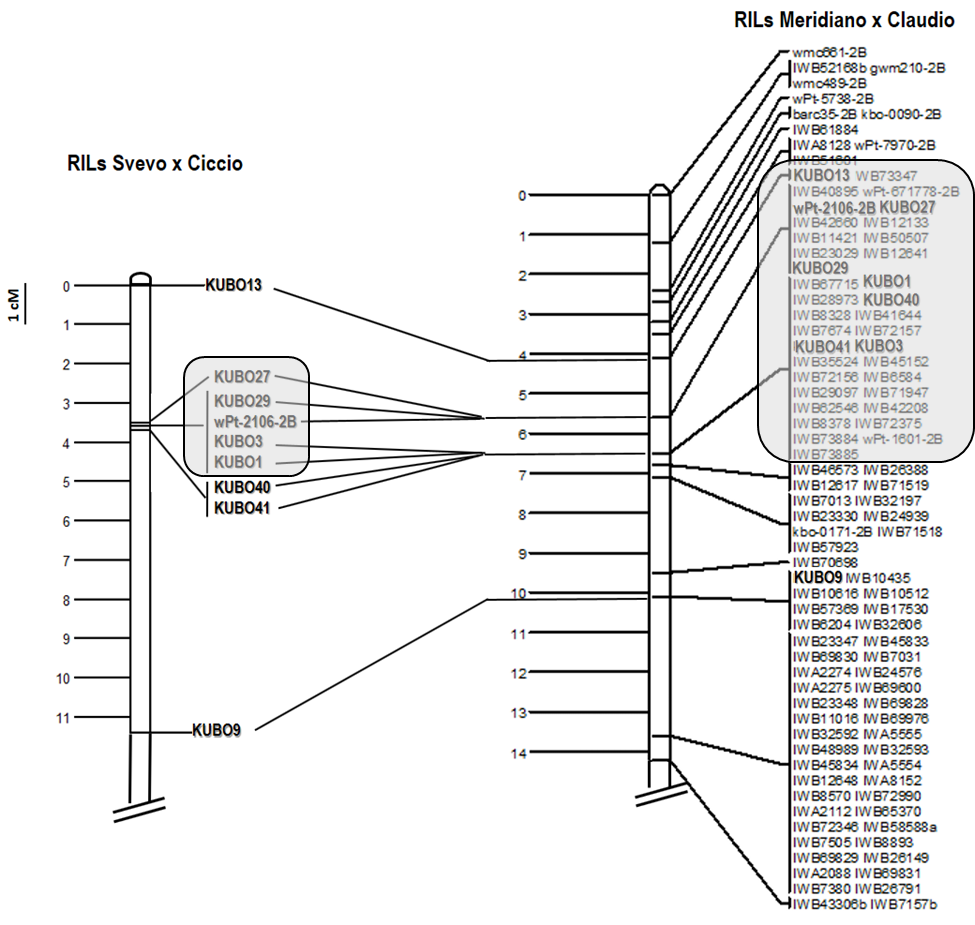


**Figure S5.** Genetic maps of RIL populations Sv × Cc and Mr × Cl obtained with the software JoinMap. Red squares delineate Sbm2 support interval.


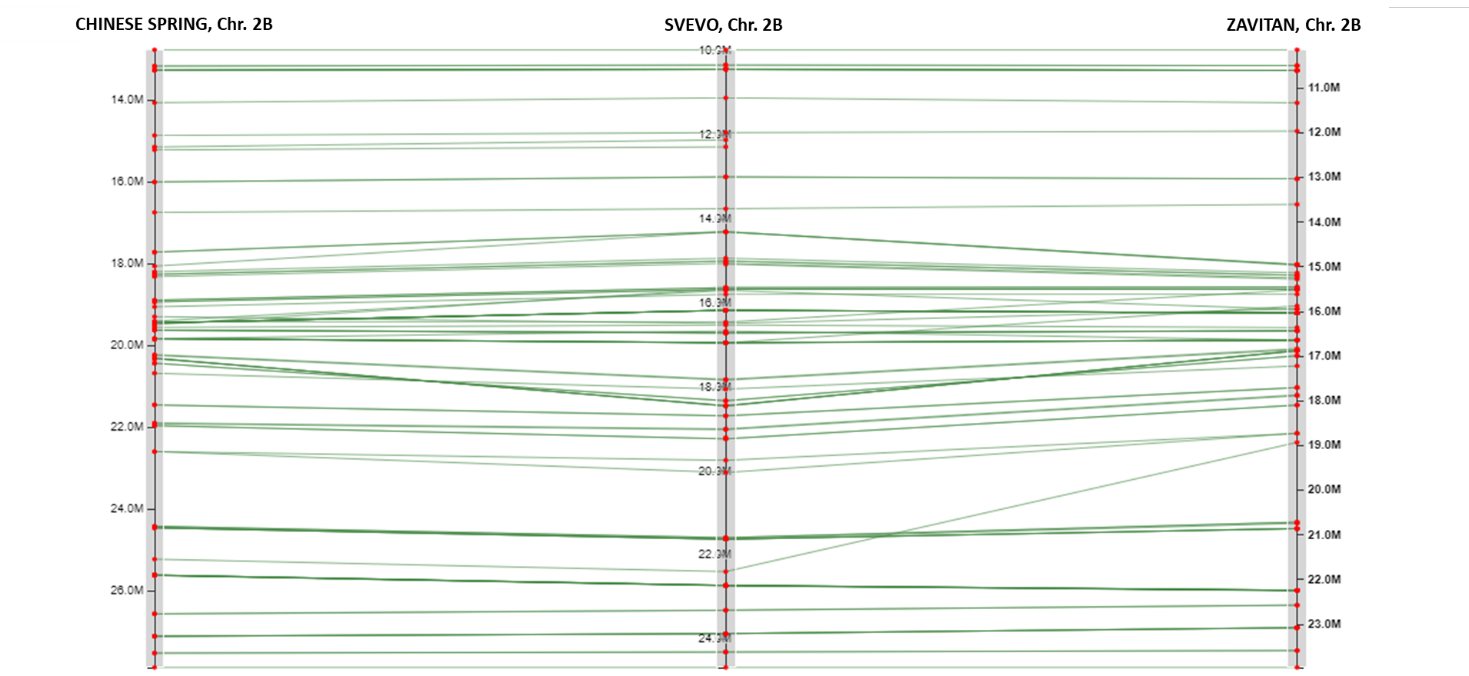


**Figure S6.** Comparison of marker order in Sbm2 region among three physical maps: Chinese Spring genome (bread wheat), Svevo genome (durum wheat) and Zavitan genome (wild emmer wheat). Markers are represented by red dots. Green lines connect markers with the same name in different genomes. Positions are expressed in bp and were retrieved blasting marker sequences against the reference genome sequences.


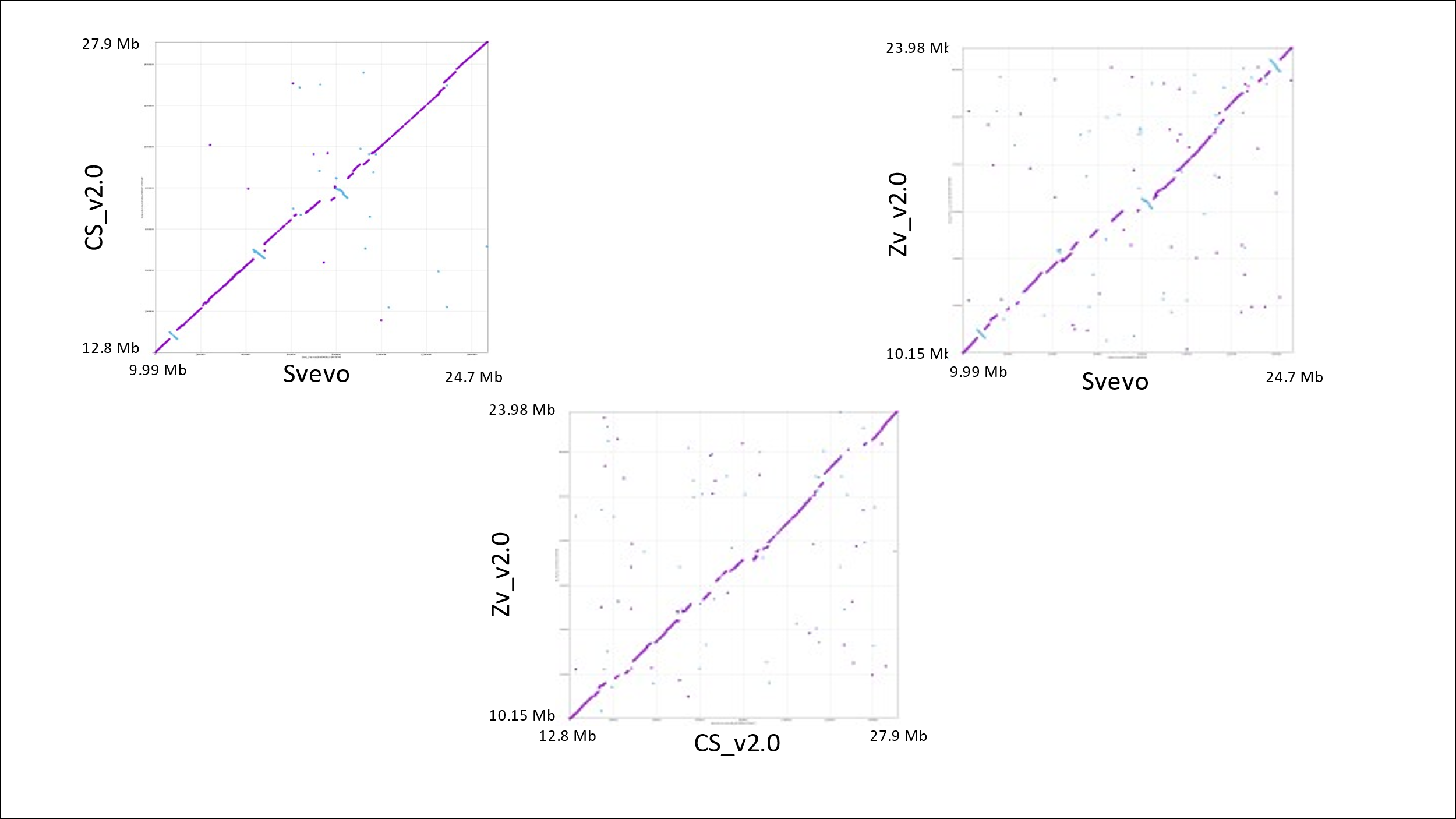


**C**

**A**

**B**

**Figure S7.** Mummer dot plot reporting the comparison between A) Chinese Spring v2.0 and Svevo , B) Zavitan v2.0 and Svevo and C) Zavitan v2.1 and Chinese Spring v2.0. The linearity between sequences is reported along the plot diagonal with purple dots, inversions are reported in light blue.


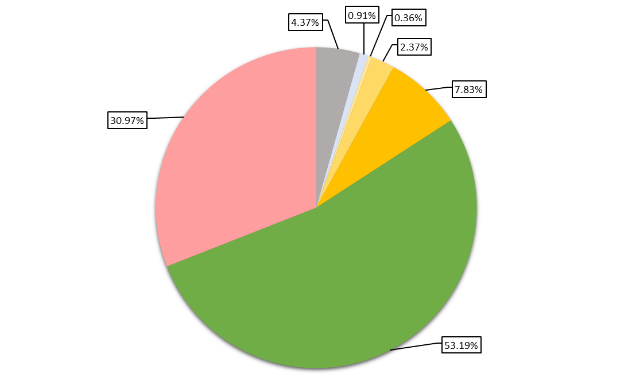

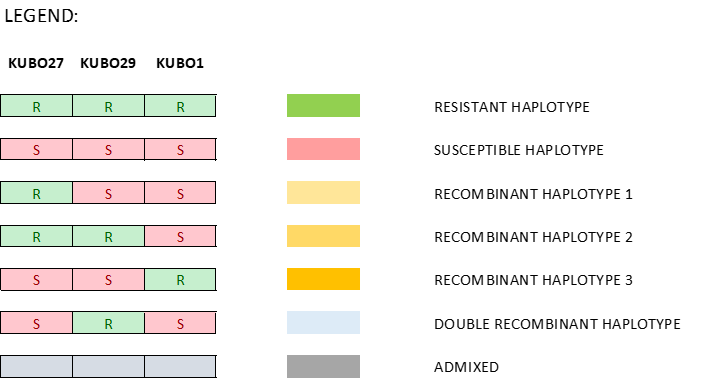


**Figure S8**: Proportion of different haplotypes at Sbm2 composing the complete panel of 549 durum wheat accessions. The haplotype refers to three markers (KUBO-27, KUBO-29 and KUBO-1) and includes the support interval of the QTL.


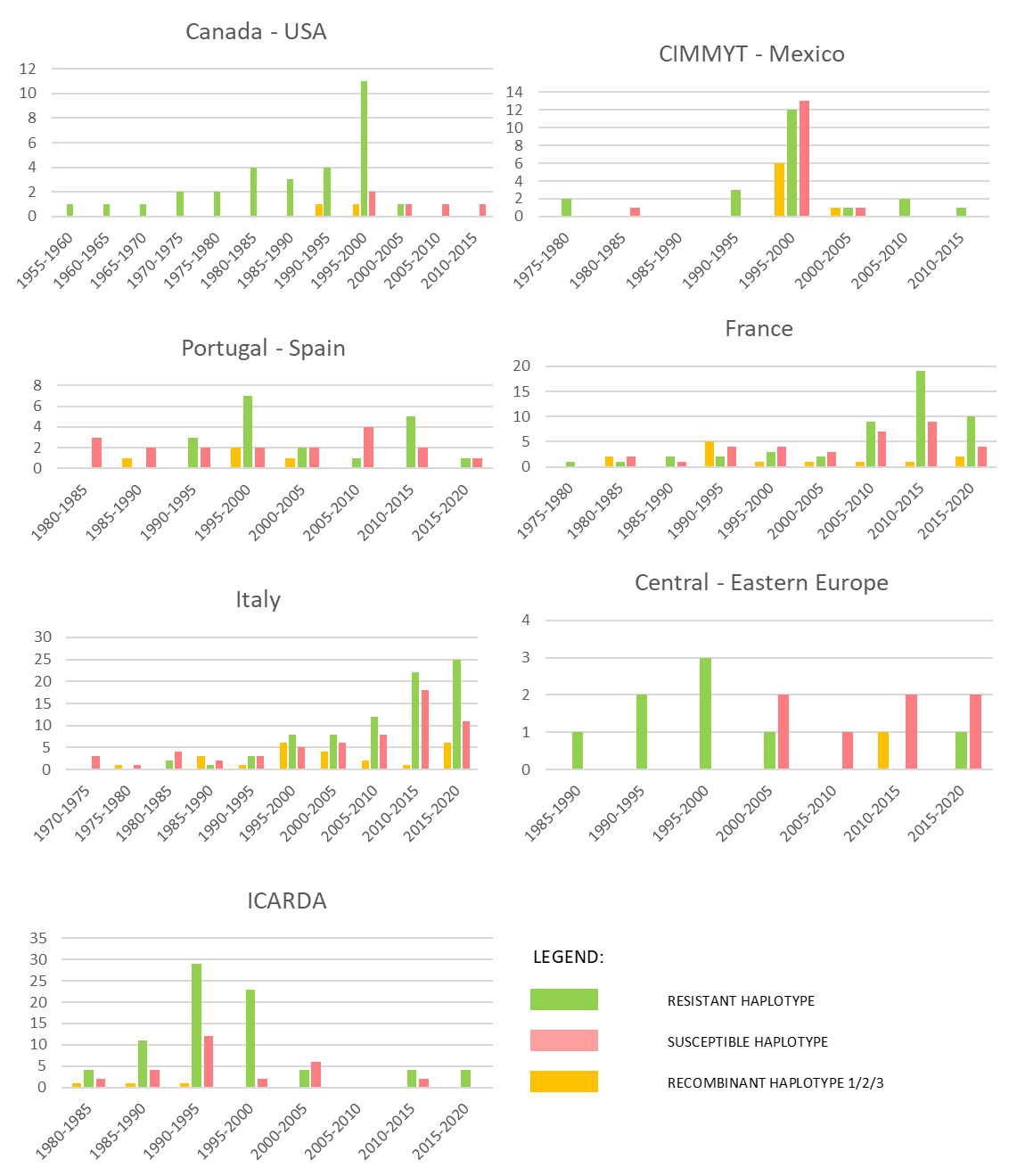


**Figure S9.** Histograms of Sbm2 haplotypes trends across intervals of 5 years based on the years of release of the accessions and on their origin. The haplotype refers to three markers (KUBO-27, KUBO-29 and KUBO-1) and includes the support interval of Sbm2. Two accessions of Central – Eastern Europe, respectively resistant and released in 1926 and recombinant and released in 1950, and three accessions of Italian origin, respectively recombinant and released in 1915 and susceptible and released in 1930 and 1940, are not reported in the histograms.

**AS**


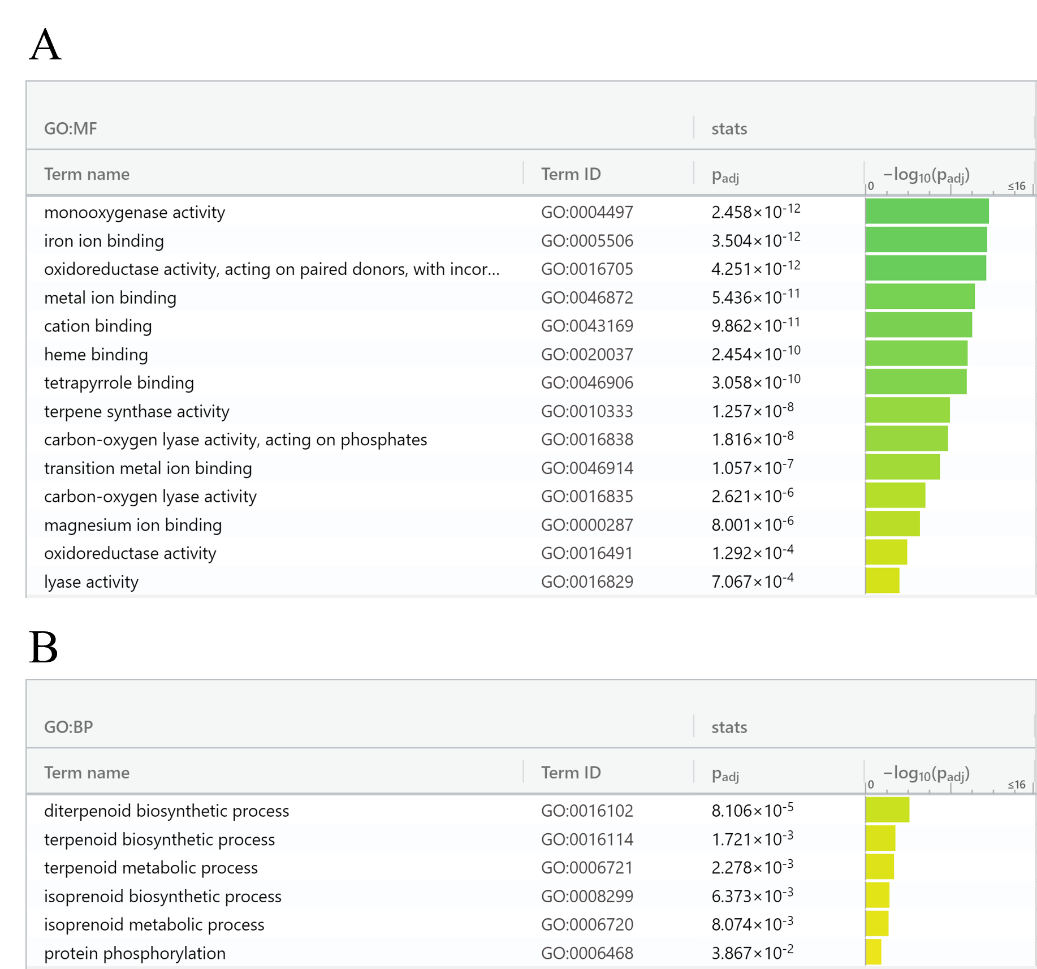


**B**

**Figure S10.** Molecular function (A) and Biological processes (B) of GO enrichment analysis. The logarithm of adjusted P value was reported for every molecular function or biological process.


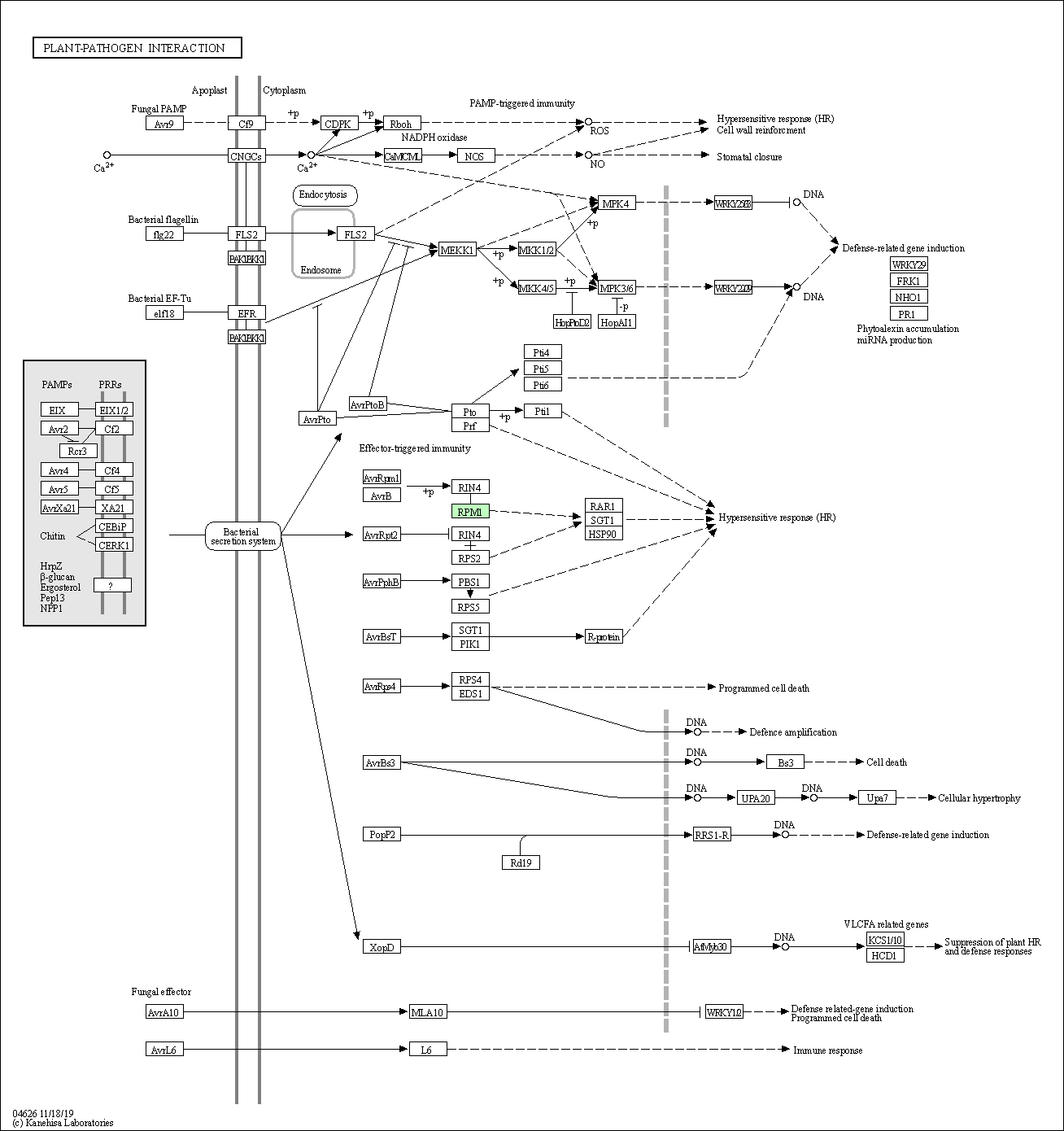


**Figure S11.** Plant-pathogen interaction pathways identified by BlastKoala analysis, corresponding to organismal system KEGG category. The gene RPM1 (TRITD2Bv1G007240) is mainly involved in the recognition of the avirulence pathogen protein (Avr) into the host organism.
